# RNA-seq and network analysis reveal unique glial gene expression signatures during prion infection

**DOI:** 10.1186/s13041-020-00610-8

**Published:** 2020-05-07

**Authors:** James A. Carroll, Brent Race, Katie Williams, James Striebel, Bruce Chesebro

**Affiliations:** Laboratory of Persistent Viral Diseases, Rocky Mountain Laboratories, National Institute of Allergy and Infectious Diseases, National Institutes of Health, 903 South Fourth Street, Hamilton, MT 59840 USA

**Keywords:** PLX5622, Scrapie, Prion, Apoptosis, Gliosis, Microglia, Astrocyte, CD11c, CD18, Neurodegeneration

## Abstract

**Background:**

Prion diseases and prion-like disorders, including Alzheimer’s disease and Parkinson’s disease, are characterized by gliosis and accumulation of misfolded aggregated host proteins. Ablating microglia in prion-infected brain by treatment with the colony-stimulating factor-1 receptor (CSF-1R) inhibitor, PLX5622, increased accumulation of misfolded prion protein and decreased survival time.

**Methods:**

To better understand the role of glia during neurodegeneration, we used RNA-seq technology, network analysis, and hierarchical cluster analysis to compare gene expression in brains of prion-infected versus mock-inoculated mice. Comparisons were also made between PLX5622-treated prion-infected mice and untreated prion-infected mice to assess mechanisms involved in disease acceleration in the absence of microglia.

**Results:**

RNA-seq and network analysis suggested that microglia responded to prion infection through activation of integrin CD11c/18 and did not adopt the expression signature associated with other neurodegenerative disease models. Instead, microglia acquired an alternative molecular signature late in the disease process. Furthermore, astrocytes expressed a signature pattern of genes which appeared to be specific for prion diseases. Comparisons were also made with prion-infected mice treated with PLX5622 to assess the impact of microglia ablation on astrocyte gene expression during prion infection. In the presence of microglia, a unique mix of transcripts associated with A1- and A2-reactive astrocytes was increased in brains of prion-infected mice. After ablation of microglia, this reactive astrocyte expression pattern was enhanced. Thus, after prion infection, microglia appeared to decrease the overall A1/A2-astrocyte responses which might contribute to increased survival after infection.

**Conclusions:**

RNA-seq analysis indicated dysregulation of over 300 biological processes within the CNS during prion disease. Distinctive microglia- and astrocyte-associated expression signatures were identified during prion infection. Furthermore, astrogliosis and the unique astrocyte-associated expression signature were independent of microglial influences. Astrogliosis and the unique astrocyte-associated gene expression pattern were increased when microglia were ablated. Our findings emphasize the potential existence of alternative pathways for activating the A1/A2 paradigm in astrocytes during neurodegenerative disease.

## Background

Many neurodegenerative diseases are characterized by the accumulation of misfolded host proteins that form aggregates in the central nervous system (CNS) [1]. Though the specific protein(s) that misfold and aggregate differ depending on the disease, it is theorized that neurodegenerative disorders such as Alzheimer’s disease, Parkinson’s disease, frontotemporal dementia, and prion disease are propagated in the CNS by an analogous mechanism known as “seeded polymerization.” Seeded polymerization stipulates that one misfolded aggregate acts as a template or “seed” to misfold other normally folded molecules of the same protein leading to self-aggregation [1, 2]. Because of the similarities in pathology and misfolded protein aggregation among the infectious prion diseases and several non-infectious neurological diseases [3], there has been a renewed interest in studying prion-like effects in many neurodegenerative diseases.

Prion disorders are transmissible, slowly progressive, usually fatal brain diseases [4]. The clinical features of prion diseases can vary, but common hallmarks in CNS are deposition of abnormally folded protease-resistant prion protein (PrPres or PrPSc), glial activation, vacuoles in the gray matter, neuroinflammation, and neurodegeneration [4–7]. Furthermore, several aspects of prion disease, such as prominent astrogliosis and microgliosis, are shared with many neuroinflammatory and neurodegenerative disorders [8–12].

Previously, we found that prion disease was accelerated by ablation of microglia following treatment with the CSF-1R inhibitor PLX5622 [13]. PLX5622-treated mice infected with prions accumulated significantly higher levels of PrPres relative to untreated mice, and PLX5622-treated mice succumbed to the disease between 20 to 32 days earlier than untreated mice depending on the prion strain. This resulted in a 20% reduction in survival time when microglia were ablated. Thus, microglia are important in reducing the amount of PrPres in the brain and are likely beneficial to the host during prion disease.

Microglia have been shown to adopt a disease-associated transcriptional phenotype (MGnD) in several neurodegenerative disease models [14]. MGnD microglia are characterized by the reduction of many homeostatic microglial genes and the concomitant upregulation of several inflammatory genes. However, microglial activation can also have a profound effect on astrocytes [15–17]. Exposure of microglia to lipopolysaccharide (LPS) elicits a pro-inflammatory microglial phenotype and promotes the conversion of quiescent astrocytes to an A1-reactive astrocytic phenotype defined by a specific transcriptional signature [16, 18]. A1-reactive astrocytes can be strongly induced in vitro by the addition of just three microglia-produced immune effectors (TNFa, IL-1a, and C1q) to the medium [16]. Moreover, A1-reactive astrocytes produced in this manner secrete a proteinaceous toxin that kills a large assortment of neurons and oligodendrocytes. Thus, A1-reactive astrocytes appear to demonstrate harmful properties, which might damage neurons and oligodendroglia in vivo.

A second reactive astrocytic phenotype, termed A2, was induced by ischemic tissue damage [16, 18]. A2-reactive astrocytes are proposed to be neuroprotective, since they upregulate many neurotrophic factors that promote neuronal survival and tissue repair. Though A2-reactive astrocytes exhibit beneficial properties, the mechanism of their induction and the influence of microglia, if any, in the process are unclear. Whether the A1 and A2 phenotypes are polar extremes in astrocyte activation states, or merely two states of a continuum is currently unknown [15]. A third gene subset described as Pan-reactive genes includes *Gfap*, *Vim*, and *Cxcl10.* These Pan-reactive genes are expressed at similar levels by both A1- and A2-reactive phenotypes and appear to be universal markers of astrogliosis [16]. In a recent report, a subset of A1- and A2-associated genes were analyzed during prion infection. Their results suggested a mixed astrocytic response with an abundance of complement component C3 expressing astrocytes in the brains of prion-infected mice [19].

Herein, we performed a longitudinal study to identify differences in gene transcripts between the brains of prion-infected and uninfected mice using RNA-seq. Our goal was to identify potential cellular networks that are altered in the CNS during disease. To investigate the role of microglia in this process, we evaluated differences in RNA profiles between prion-infected mice that were untreated or PLX5622-treated to ablate microglia from the CNS [13, 20, 21]. Both comparisons yielded numerous alterations in biological, cellular, and functional processes. In addition, there was little evidence of a MGnD phenotypic response and astrocytes expressed a unique signature comprised of several A1- and A2-associated genes during prion disease. In contrast to recent reports, we observed increased expression of A1−/A2-astrocyte-associated genes in prion-infected mice in the absence of microglia. This suggested that activation of astrocytes can occur independent of microglial influences.

## Methods

### Ethics statement

All mice were housed at the Rocky Mountain Laboratories (RML) in an AAALAC-accredited facility in compliance with guidelines provided by the Guide for the Care and Use of Laboratory Animals (Institute for Laboratory Animal Research Council). Experimentation followed RML Animal Care and Use Committee approved protocol 2016–043-E.

### Normal brain and scrapie inoculations/infections

C57BL/10 mice were originally obtained from Jackson Laboratories and have been inbred at RML for several years. Six to eight-week-old male and female mice were inoculated intracranially (ic) with 30 μl of either 1.0% normal brain homogenate (NBH) or 1.0% RML scrapie brain stock. The RML scrapie stock was titered previously in C57 mice and determined to contain 2.4 × 10^4^ ID50 in each 30 μl volume [13]. At selected time points post-inoculation (Additional File, Table S1), mice were euthanized by isoflurane anesthesia overdose followed by cervical dislocation. Brains were removed and half was used for RNA isolation and analysis.

### PLX5622 treatment

Mice were fed purified rodent diet AIN-76A (D10001, Research Diets, Inc.) with or without supplementation with compound PLX5622 (1200 ppm, kindly provided by Plexxikon Inc., Berkeley, CA). This concentration of PLX5622 in rodent chow has been shown to reduce microglia in the brain by approximately 80% within 7 days and approximately 90% within 21 days of administration [22]. Six to eight-week-old male and female mice were fed control chow for 14 days after inoculation with prions or NBH to allow mice to convalesce [13]. Then treated mice were switched to PLX5622 supplemented chow and maintained on this diet until euthanized at specific times or at the experimental endpoint (Additional File, Table S1).

### RNA isolation

Total RNA was isolated from the left hemisphere of mouse brain by dissociation of tissue in 2 ml TRI-reagent (Sigma) following the manufacturer’s protocol and pelleted by centrifugation (12,000×g, 15 min, 4 °C). Isolated RNA was rinsed in 2 ml 75% ethanol, centrifuged for 10 min at 13,000 x g, and air dried. Total RNA was suspended in 100 μl of DNase reaction buffer (Ambion) and digested with 6 units of DNase I (Ambion) of 30 min at room temperature. RNA was re-isolated and cleaned using RNA Clean & Concentrator-25 column kit (Zymo Research), eluted with 150 μl nuclease-free water with 1 x RNase Inhibitor (SUPERase-In, Ambion), and stored at − 80 °C until use.

### RNA-seq analysis

RNA-seq was performed by LC Sciences from RNA isolated from the brains of 47 mice. The number of mice per group can be found in Additional File, Table S1. Total RNA quantity and purity were analyzed using a Bioanalyzer 2100 and with an RNA 6000 Nano LabChip Kit (Agilent, CA, USA). All RNA samples had a RIN (RNA Integrity Number) value > 7.0. Approximately 10 μg of total RNA was used to isolate Poly (A) mRNA with poly-T oligo attached magnetic beads (Invitrogen). Following purification, the poly (A) + RNA fraction was fragmented into small pieces using divalent cations under elevated temperature. Cleaved RNA fragments were reverse-transcribed to create the final cDNA library in accordance with the protocol for the mRNA-Seq sample preparation kit (Illumina, San Diego, USA), the average insert size for the paired-end libraries was 300 bp (±50 bp). Paired-end sequencing of each sample was performed on an Illumina Hiseq 4000 at LC-Bio (China) following the vendor’s recommended protocol.

For transcript assembly, programs Cutadapt and perl scripts were used to remove the reads that contained adaptor contamination, low quality bases, and undetermined bases [23]. Then sequence quality was verified using FastQC (http://www.bioinformatics.babraham.ac.uk/projects/fastqc/). HISAT2 was used to map reads to the genome of *Mus musculus* [24]. The mapped reads of each sample were assembled using StringTie [25]. Then, all transcriptomes were merged to reconstruct a comprehensive transcriptome using perl scripts and gffcompare. After the final transcriptome was generated, StringTie [25] and Ballgown [26] were used to estimate the expression levels of all transcripts.

To estimate differences in gene expression, the program StringTie was used to evaluate the expression levels for mRNAs by calculating the fragments per kilobase of transcript per million mapped reads (FPKM) [25]. The differentially expressed mRNAs were selected with log2 (fold change) > 1 or log2 (fold change) < − 1 and with statistical significance (*p* value < 0.05) by R package Ballgown [26]. Gene Ontology (GO) enrichment analysis was performed using the online platform provided by the GO Consortium (http://geneontology.org). A Pearson correlation heatmap and average linkage hierarchical cluster analysis were generated using our RNA-seq FPKM values for the described Pan-, A1-, and A2-reactive astrocyte genes and for the genes associated with MGnD microglia using the web-enabled application Heatmapper [27]. Network analysis using STRING version 11.0 [28] was performed on the top 57 genes predominantly expressed by microglia that were significantly increased by ≥3.0-fold in the brain during prion infection. The STRING network analysis parameters were set to experimental and database interaction sources only with the highest confidence (0.9). STRING network clustering was performed using Markov clustering with an inflation parameter setting of 3.5.

We assigned the predominant basal gene expression to mature microglia, neurons, astrocytes, and/or oligodendrocytes by consulting Zhang et al. [29] and entering each gene of interest into the on-line tool provided at brainrnaseq.org. Genes for which no single cell population displayed most of the transcripts by > 2.0-fold were labeled according to the multiple cell types responsible for its transcription. The term “All” refers to microglia, neurons, astrocytes, and oligodendrocytes. We did not differentiate between “newly formed oligodendrocytes” and “myelinating oligodendrocytes” in this analysis but refer to them collectively as “oligodendrocyte.”

### In situ hybridization in formalin fixed brain tissues

For in situ hybridization studies, C57BL/6 mice and GFAP-deficient mice (B6;129S-Gfap^tm1Mes^/J, Stock number 002642, Jackson Laboratories) were inoculated with prions and allowed to progress until clinical, when they were euthanized by isoflurane anesthesia overdose followed by cervical dislocation. The brains were removed and immediately placed in 10% neutral buffered formalin (NBF) for 3 to 5 days and then embedded in paraffin. Four micrometer sections were cut from the tissue samples using a standard Leica microtome, placed on positively charged glass slides, and air-dried overnight at room temperature. Slides were baked at 60 °C for 1 h and stored at room temperature for no more than 1 week prior to hybridization.

For colorimetric in situ hybridization (CISH), slides with 4 um sagittal brain sections were deparaffinized with xylene by incubation 3 times for 5 min each. Slides were then washed twice in 100% ethanol or 5 min each and allowed to air dry. Slides were then prepared for dual CISH using the protocol specified in the ViewRNA ISH Tissue 2-Plex assay kit manufacture by Affymetrix. Briefly, slides were heat treated (90 °C, 10 min), rinsed twice with double-distilled water, and placed in PBS. Samples then underwent protease digestion with 400 μl of Protease QF (supplied by manufacturer) for 10 min at 40 °C. Samples were then washed twice with PBS (1 min each wash) and fixed in 10% NBF at room temperature for 5 min. Samples were rinsed twice with PBS (1 min each wash) and hybridized with probes specific to *Cxcl10* (type 6 probe) and to *Gfap* (type 1 probe) according to the manufactures’ protocol. The *Cxcl10* type 6 probe hybridization was detected using Label probe 6-alkaline phosphatase (40 °C, 15 min), rinsed 3 times in PBS, and visualized using fast blue substrate for 30 min at room temperature in the dark. The reaction was halted using the provided stopping solution (30 min, room temperature) and rinsed twice in PBS for 1 min per rinse. The *Gfap* type 1 probe was similarly detected using Label probe 1-alkaline phosphatase (40 °C, 15 min), rinsed 3 times in PBS, and visualized using fast red substrate for 30 min at room temperature in the dark. Slides were then washed in PBS and counter stained with Gill’s hematoxylin. Slides were air dried and mounted for imaging.

### qRT-PCR analysis

For quantitative analysis of changes in transcription using qRT-PCR, 400 ng of high-quality RNA from each sample was reverse transcribed to synthesize cDNA using the RT^2^ First Stand Kit per manufacturer’s instructions (Qiagen). Each cDNA reaction was mixed with 2x RT2 SYBR Green Mastermix purchased from Qiagen with RNase-free water to a final volume of 1.3 ml. Ten microliters of the mixture was then added to each well of a 384-well format plate for analysis. All validated primer sets were purchased from Qiagen. The analysis was carried out on an Applied Biosystems ViiA 7 Real-Time PCR System with a 384-well block using the following conditions: 1 cycle at 10 min, 95 °C; 40 cycles at 15 s, 95 °C then 1 min, 60 °C with fluorescence data collection. Melting curves were generated at the end of the completed run to determine the quality of the reaction products. Raw threshold cycle (C_T_) data was collected with a C_T_ of 35 as the cutoff. All C_T_ values were normalized to the average of the C_T_ values for the housekeeping genes *Actb*, *Gapdh*, and *Hsp90ab1*. Changes in transcription were calculated by using the ΔΔC_T_ based method [30]. Statistical analysis was performed using the unpaired Student’s t-test to compare the replicate ΔC_T_ values for each gene in the control group versus infected groups. A mean of ≥2.0-fold change and *p*-value of ≤0.05 considered significant. For qRT-PCR data, *p*-values were not adjusted for multiple comparisons since we were interested in only controlling for the individual error rate, where an adjustment for multiple tests is deemed unnecessary.

### Immunohistochemical detection microglia and astroglia

Portions of brain were removed and placed in 10% neutral buffered formalin for 3 to 5 days. Tissues were then processed and embedded in paraffin. Sections were cut using a standard Leica microtome, placed on positively charged glass slides, and air-dried overnight at room temperature. The following day, slides were heated in an oven at 60 °C for 30 min. Deparaffinization, antigen retrieval, and staining were performed using the Ventana automated Discovery XT stainer.

To stain microglia, rabbit anti-Iba1 was used at a 1:2000 dilution and was a gift from John Portis, RML, Hamilton, MT. To stain astrocytes, rabbit anti-glial fibrillary acidic protein (GFAP) (Dako number Z0334) was used at a dilution of 1:3500. Primary antibodies were diluted in PBS containing stabilizing protein and 0.1% Proclin 300 (Ventana antibody dilution buffer). Diluent without antibody was used as a negative control. The Ventana streptavidin-biotin alkaline phosphatase system was used to detect Iba1 and GFAP [31] with the exceptions that the secondary antibody was goat anti-rabbit Ig (HK336-9R; Biogenex) and fast red chromogen was used. Slides were examined, and photomicrographs were taken and observed using an Olympus BX51 microscope and Microsuite FIVE software. Hematoxylin was used as a counterstain for all slides. Iba1 positive cell bodies were assessed as previously described [13].

Histological scoring of GFAP was performed as follows. Coronal brain sections stained with anti-GFAP antibody were examined using scanned images of stained slides (Aperio Imagescope software). Each section was given a subjective score from 0 to 4 based on the following criteria: 0, no staining present (seen in our no primary antibody applied controls); 1, staining present primarily in white matter regions, near the edges of the tissue or associated with blood vessels, cellular GFAP staining within the cerebral cortex was rare (this pattern of staining is observed in uninfected or mock infected mice using our IHC protocol); 2, staining as described for 1 plus focal areas of GFAP staining in the cerebral cortex and a generalized increase in overall thalamic GFAP; 3, staining as described for 2 plus many areas of GFAP staining in the thalamus and cerebral cortex, occasionally confluent with adjacent foci; 4, abundant GFAP staining throughout the entire section.

## Results

### Analysis of altered transcripts during prion infection in mice by RNA-seq

To gain insight into the biological and cellular processes that are altered in the brain during prion infection, we inoculated mice with prion strain RML and isolated RNA from brains at 80 days post-inoculation (dpi) (mid-incubation), 100 dpi (preclinical), and at the clinical endpoint (average ~ 157 dpi) (Additional File, Table S1). For controls we inoculated a cohort of mice with NBH and harvested them at similar times. RNA-seq was performed yielding ≥40 million valid reads per sample resulting in similarities in the percentage of mapped reads, unique mapped reads, positive and negative strand reads, etc. The mapping statistics indicated analogous transcript representation in the constructed libraries. Furthermore, overall mapping of sequences to exon, intron, or intergenic regions were also comparable for all brain RNA samples (Additional File, Supplementary Dataset 1).

We initially assessed the variance of the RNA-seq dataset in control mice that were either PLX5622-treated or untreated at 80, 98, and 161 days after mock infection with NBH. RNA-seq expression data was analyzed by principal component analysis (PCA) (Additional File, Supplementary Dataset 1). Plotting these results indicated two important points: 1) most of the mock-infected mice that were either PLX5622-treated or untreated clustered tightly, indicative of negligible variance in overall gene expression and 2) mice treated with PLX5622 for 161 days segregated outside of this cluster, suggesting long-term PLX5622 treatment alone has a small but observable effect on overall gene expression in mice. Because we found negligible variance between the untreated NBH injected control mice at 80, 98, and 161 dpi, we group them together (*n* = 6) and used them as a control sample set for comparison with prion-infected mice.

### RNA-seq analysis of RML-infected and uninfected mice at 80 dpi

**Fig. 1:**
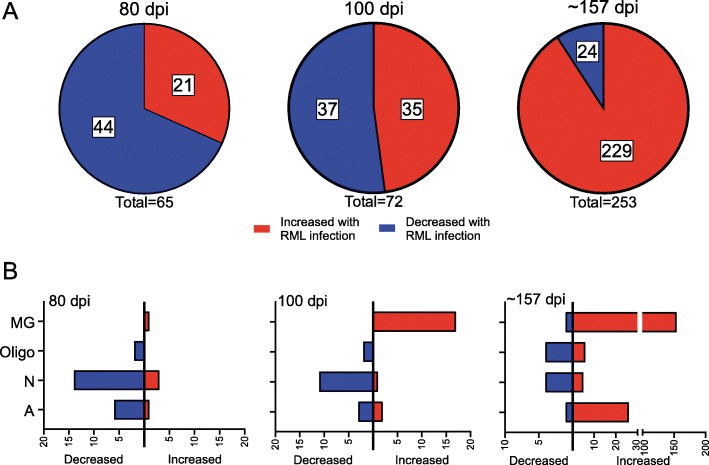
Summary of differentially expressed genes resulting from RNA-seq comparisons of RML-infected to Uninfected mice at 80 dpi, 100 dpi, and ~ 157 dpi. In panel **a**, the red indicates the proportion of genes increased in the brains of RML-infected mice, while blue represents the proportion of genes decreased in the brains of RML-infected mice. Panel **b** indicates the number of differentially expressed genes either increased (red bars) or decreased (blue bars) that are predominately expressed in the indicated cells of the CNS during basal conditions. MG refers to microglia, Oligo refers to oligodendrocytes, N refers to neurons, and A refers to astrocytes. Genes that are similarly expressed in more than one cell type are not shown for simplicity. The specific genes, their fold change, their cellular expression, and statistical analysis can be found in Additional File, Supplementary Dataset 2

Eighty dpi is approximately the mid-incubation time for infection with prion strain RML. At this time, we could detect 65 genes that were significantly altered in the brains of prion infected mice relative to control mice (Fig. 1a). The comprehensive list of differentially expressed genes at 80-, 100-, and ~ 157-dpi is provided in Additional File, Supplementary Dataset 2. Of these altered genes, 44 were decreased, while 21 were increased. Three genes that were decreased (*Ddx3y*, *Eif2s3y*, and *Uty*) are Y-linked and identified as altered in our analysis due to sex differences between the cohorts and were not considered further.

**Fig. 2:**
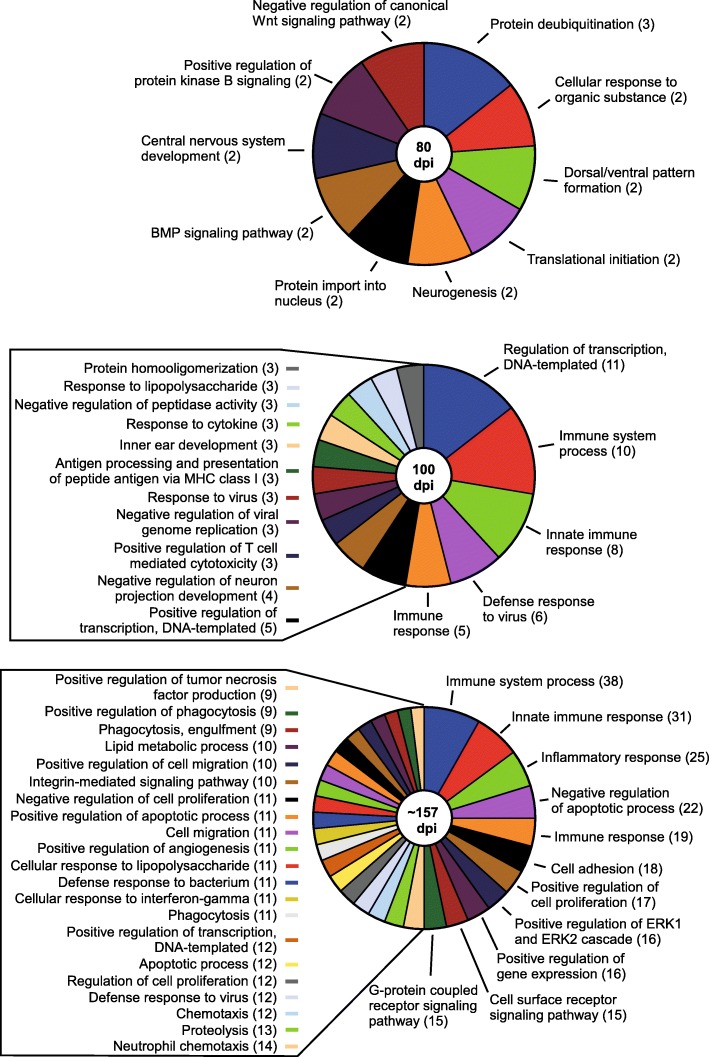
Itemization of the GO: Biological Processes that were statistically enriched when comparing RML-infected mice to Uninfected mice by RNA-seq at 80, 100, and ~ 157 dpi. Few GO biological categories were affected at 80 dpi, but evidence of dysregulation in more biological processes was apparent as prion disease progressed. The affected categories are indicated with the number of genes that were differential expressed that are associated with that specific biological process in parentheses. Not all enriched biological processes could be included for 100 dpi and ~ 157 dpi but can be found in Additional File, Supplementary Dataset 3, where a full analysis of GO: Biological Processes, Cellular Component, and Molecular Function and the associated genes can be found

Most of the genes identified as altered at 80 dpi during prion infection are predominantly expressed by astrocytes (i.e. *Irs2, Timp3*, and *Kdm5d*), oligodendrocytes (i.e. *Lrrn3, Mal*, and *Hmgb1*), and neurons (i.e. *Fem1b, Irf2bpl, Bhlhe22, Bex2, Ndn*, and *Sprn*) (Fig. 1b, Additional File, Supplementary Dataset 2). Gene Ontology (GO) enrichment analysis of altered genes [32] was performed to reduce complexity and highlight the Biological Processes, Cellular Components, and Molecular Functions potentially influenced during prion disease. The comprehensive GO analysis complete with altered genes and associated categories for 80-, 100-, and ~ 157-dpi can be found in Additional File, Supplementary Dataset 3. From our perspective, the GO: Biological Processes category offered more relevant information, and a summary is presented Fig. 2. Ten statistically significant Biological Process subcategories with two or more genes represented were enriched in the 80-dpi data set. These included processes such as Protein deubiquitination (*Josd1*, *Usp22*, and *Usp29*), Neurogenesis (*Foxg1* and *Bhlhe22*) and Nervous system development (*Ndn* and *Nog*) that might influence the early stages of prion pathogenesis.

### RNA-seq analysis of RML-infected and uninfected mice at 100 dpi

At 100dpi, which is a preclinical time in mice infected with strain RML, we observed 72 changes in gene transcripts when comparing infected and uninfected mice with a near equal split in the number of increased and decreased genes (Fig. 1a and Additional File, Supplementary Dataset 2). We observed several genes that increased > 4-fold in the brain in prion-infected mice that are primarily expressed in astrocytes (*Jun*, *Cxcl10*, and *Klf9*) or neurons (*Irf2pb1* and *Klf9*). Many genes indicative of microglial activation and/or proliferation in the brain (i.e. *Lag3, Cst7, Clec7a, Itgax, Trem2,* and *Fcrls*) were also increased at 100 but not at 80 dpi (Fig. 1b and Additional File, Supplementary Dataset 2). Furthermore, several Pan- and A1-reactive astrocyte associated genes (*Cxcl10, Gfap, Serpina3n, C4b, Ifitm3/6, H2-D1,* and *B2m*) (Table 1) were also up-regulated in prion inoculated mice, suggesting evidence of the conversion of quiescent astrocytes to a potential A1 phenotype in the preclinical brain.

**Table 1:** Analysis of Pan, A1, and A2 astrocyte signature gene expression by RNA-seq comparing RML prion-infected mice to uninfected mice

Gene	Description	80 dpi^a^		100 dpi		~ 157^c^ dpi	
		FC^b^	*P* value	FC	*P* value	FC	*P* value
Pan							
*Aspg*	Asparaginase	1.1	4.7 × 10^− 2^	1.4	7.2 × 10^− 4^	**3.1**	**3.8 × 10**^**− 10**^
*Cd44*	CD44 antigen	1.1	4.7 × 10^− 2^	1.2	8.9 × 10^− 3^	**3.0**	**6.3 × 10**^**− 11**^
*Cxcl10*	Chemokine (C-X-C motif) ligand 10	**2.4**	**2.1 × 10**^**− 3**^	**4.0**	**1.0 × 10**^**− 5**^	**4.4**	**4.1 × 10**^**− 6**^
*Gfap*	Glial fibrillary acidic protein	1.4	3.6 × 10^− 2^	**3.3**	**5.5 × 10**^**− 6**^	**12.0**	**6.1 × 10**^**− 11**^
*Gfap*	Splice variant id MSTRG.4106	1.2	8.5 × 10^− 3^	*1.5*	*3.6 × 10*^*− 3*^	**4.3**	**1.7 × 10**^**− 8**^
*Gfap*	Splice variant id MSTRG.4107	1.0	6.1 × 10^− 1^	1.1	1.4 × 10^− 1^	*1.9*	*5.0 × 10*^*− 6*^
*Hspb1*	Heat shock protein 1	0.9	2.4 × 10^− 1^	1.1	6.6 × 10^− 1^	**3.3**	**4.1 × 10**^**− 7**^
*Lcn2*	Lipocalin 2	1.0	7.1 × 10^− 1^	1.2	1.8 × 10^− 1^	**4.9**	**6.9 × 10**^**− 9**^
*Osmr*	Oncostatin M receptor	1.1	2.2 × 10^− 1^	1.2	5.4 × 10^− 2^	**3.6**	**6.1 × 10**^**− 7**^
*Serpina3n/3f/3 m*	Serine (or cysteine) peptidase inhibitor, clade A, member 3 N /3F/ 3 M	1.3	6.2 × 10^− 2^	**2.9**	**5.1 × 10**^**− 6**^	**12.3**	**8.3 × 10**^**− 11**^
*Timp1*	Tissue inhibitor of metalloproteinase 1	1.2	2.6 × 10^− 1^	1.2	2.7 × 10^− 2^	**2.4**	**9.8 × 10**^**− 7**^
*Vim*	Vimentin	0.8	1.1 × 10^− 1^	1.2	2.0 × 10^− 1^	**4.3**	**2.8 × 10**^**− 7**^
A1							
*A2m*	Alpha-2-macroglobulin	1.2	3.5 × 10^− 2^	1.4	1.6 × 10^− 2^	**4.8**	**1.1 × 10**^**− 9**^
*B2m*	Beta-2 microglobulin	1.2	4.6 × 10^− 1^	**2.2**	**9.7 × 10**^**− 4**^	**3.7**	**1.9 × 10**^**− 6**^
*C3*	Complement component 3	0.9	4.2 × 10^− 1^	1.2	3.3 × 10^− 2^	**4.2**	**2.2 × 10**^**− 9**^
*C4b*	Complement component 4B (Chido blood group)	*1.5*	*4.9 × 10*^*− 4*^	**2.8**	**4.2 × 10**^**− 7**^	**7.4**	**1.3 × 10**^**− 13**^
*Chil1 (Chi3l1)*	Chitinase-Like 1 (Chitinase 3-Like 1)	1.2	5.2 × 10^− 2^	*1.6*	*4.3 × 10*^*− 4*^	**2.5**	**3.1 × 10**^**− 8**^
*Fbln5*	Fibulin 5	0.9	3.6 × 10^− 3^	1.0	8.3 × 10^− 1^	*1.6*	*1.9 × 10*^*− 5*^
*Gbp2*	Guanylate binding protein 2	1.0	6.8 × 10^− 1^	1.4	8.0 × 10^− 3^	**2.1**	**2.0 × 10**^**− 5**^
*Gbp3*	Guanylate binding protein 3	1.2	2.4 × 10^− 1^	*1.5*	*1.5 × 10*^*− 2*^	**2.0**	**2.5 × 10**^**− 4**^
*Gbp6/10*	Guanylate binding protein 6/10	1.1	4.4 × 10^− 1^	1.4	9.8 × 10^− 4^	*1.6*	*6.2 × 10*^*− 5*^
*Ggta1*	Glycoprotein galactosyltransferase alpha 1, 3	0.9	2.1 × 10^− 1^	0.9	3.3 × 10^− 1^	*1.7*	*4.1 × 10*^*− 3*^
*H2-D1*	Histocompatibility 2, D region locus 1	1.3	2.5 × 10^− 2^	**2.3**	**8.3 × 10**^**− 5**^	**3.5**	**9.8 × 10**^**− 8**^
*H2-T23*	Histocompatibility 2, T region locus 23	1.0	9.8 × 10^− 1^	*1.6*	*3.1 × 10*^*− 4*^	**2.0**	**5.3 × 10**^**− 6**^
*Ifitm3/6*	Interferon induced transmembrane protein 3/6	2.0	7.1 × 10^− 2^	**2.5**	**7.5 × 10**^**− 3**^	**2.7**	**1.9 × 10**^**− 2**^
*Igtp*	Interferon gamma induced GTPase	1.1	1.6 × 10^− 1^	*1.5*	*1.3 × 10*^*− 4*^	*1.8*	*2.4 × 10*^*− 6*^
*Iigp1*	Interferon inducible GTPase 1	1.1	7.0 × 10^− 1^	1.3	3.7 × 10^− 2^	*1.6*	*8.7 × 10*^*− 4*^
*Irgm1*	Immunity-related GTPase family M member 1	1.2	1.7 × 10^− 1^	*1.6*	*2.6 × 10*^*− 3*^	**2.2**	**5.2 × 10**^**− 6**^
*Psmb8*	Proteasome (prosome, macropain) subunit, beta type 8	1.1	6.3 × 10^− 1^	*1.6*	*1.3 × 10*^*− 2*^	**2.6**	**2.9 × 10**^**− 5**^
*Serping1*	Serine (or cysteine) peptidase inhibitor, clade G, member 1	0.8	9.5 × 10–2	1.3	2.7 × 10^−2^	**2.1**	**1.0 × 10**^**− 6**^
*Slc22a4*	Solute carrier family 22 (organic cation transporter), member 4	1.0	9.3 × 10–1	1.1	1.3 × 10^−2^	*1.5*	*6.9 × 10*^*−5*^
A2							
*Anxa2*	Annexin A2	0.8	8.1 × 10^−2^	1.0	9.7 × 10^−1^	*1.6*	*1.3 × 10*^*− 4*^
*Anxa3*	Annexin A3	1.0	9.0 × 10^−1^	1.2	6.3 × 10^− 2^	**2.5**	**2.1 × 10**^**−6**^
*Cd14*	CD14 antigen	1.1	4.5 × 10^−1^	1.4	1.3 × 10^−2^	**2.8**	**9.4 × 10**^**−8**^
*Cd109*	CD109 antigen	1.0	7.5 × 10^−1^	1.0	5.4 × 10^− 1^	**2.1**	**3.5 × 10**^**− 9**^
*Emp1*	Epithelial membrane protein 1	0.9	2.6 × 10^−1^	1.1	1.2 × 10^− 1^	*1.7*	*6.7 × 10*^*−7*^
*Flnc*	Filamin C, gamma	1.1	2.9 × 10^−1^	1.2	1.2 × 10^−3^	**2.4**	**4.3 × 10**^**−10**^
*Hmox1*	Heme oxygenase 1	1.1	4.9 × 10^−1^	*1.5*	*6.8 × 10*^*−4*^	**2.7**	**1.3 × 10**^**−7**^
*Icam1/4*	Intercellular adhesion molecule 1/4	1.0	6.1 × 10^−1^	1.2	3.2 × 10^−4^	*1.7*	*1.6 × 10*^*−8*^
*Lgals3*	Lectin, galactose binding, soluble 3	1.0	8.6 × 10^−1^	1.3	9.7 × 10^−3^	**4.0**	**8.4 × 10**^**−7**^
*Sbno2*	Strawberry notch homolog 2	1.2	5.1 × 10^−2^	1.2	2.6 × 10^− 2^	**2.0**	**4.4 × 10**^**−7**^
*S1pr3*	Sphingosine-1-phosphate receptor 3	1.3	2.0 × 10^−1^	*1.5*	*1.5 × 10*^*−2*^	**4.1**	**8.4 × 10**^**−7**^
*Tgm1*	Transglutaminase 1, K polypeptide	1.1	4.5 × 10^−3^	1.1	1.2 × 10^−2^	**3.2**	**4.2 × 10**^**−8**^
*Tnfrsf12a*	Tumor necrosis factor receptor superfamily, member 12a	1.2	1.6 × 10^−1^	1.3	3.2 × 10^− 1^	**2.1**	**1.5 × 10**^**−5**^

Bolded values denote genes increased ≥2.0-fold with *p* values ≤0.05 (5.0 × 10^− 2^) in RML-infected mice

Values in italics are increased between 1.5-fold and 1.9-fold with *p* values ≤0.05 (5.0 × 10^− 2^) in RML-infected mice

^a^*dpi* days post inoculation

^b^*FC* fold change

^c^ ~ 157 is the average dpi to clinical endpoint for RML-infected mice

Similar to 80 dpi, most of the genes that were decreased at 100 dpi in the prion-infected brain are mainly expressed by astrocytes, neurons, and oligodendrocytes (Fig. 1b). These included *Hmgb1, Egr1, Timp3,* and *Bex2* that were decreased at the mid-incubation time (80 dpi) as well (Additional File, Supplementary Dataset 2). Also decreased at 100 dpi were the genes *Lgi1* and *Lin7c*, which are predominately expressed in neurons [29] and function in regulating voltage-gated potassium channels [33] and in localizing synaptic vesicles at synapses [34], respectively.

At 100 dpi, there were over 30 GO: Biological Processes statistically enriched with 2 or more genes represented. Most notable were processes that dealt broadly with various aspects of the immune system, comprising > 44% of the genes determined to be increased in the brain during preclinical prion infection (Fig. 2). Genes involved in Negative regulation of neuron projection development (*B2m, Gfap, H2-D1,* and *H2-K1*) (Fig. 2) were increased at this time, indicating dysregulation in neurite production. In addition to several Biological Processes, there was an enrichment of altered genes involved with the GO: Cellular Component of the Synapse (*C4b, Frrs1l, Lgi1, Lin7c*, and *Shisa7*) (Additional File, Supplementary Dataset 3), indicating potential disruption of normal electrochemical signaling between neurons in the preclinical prion-infected brain.

### RNA-seq analysis of RML-infected and uninfected mice at clinical endpoint (~ 157 dpi)

Not surprisingly, the greatest number of differences by RNA-seq were observed when prion-infected mice at clinical endpoint (~ 157 dpi) were compared to uninfected mice (Fig. 1a and Additional File, Supplementary Dataset 2). Greater than half of the genes that were increased in prion-infected mice are proposed to be mainly expressed by microglia (154 of 229 genes) (Fig. 1b), consistent with previous reports that microglial activation is prevalent during prion disease [35, 36]. There was also a notable increase in many astrocyte genes associated with Pan- (*Serpina3n, Gfap, Lcn2, Cxcl10,* and *Vim*), A1- (*C4b, A2m, B2m, H2-D1,* and *Ifitm3/6*) and A2-reactive (*Lgals3, Tgm1, Cd14, Hmox1,* and *Anxa3*) phenotypes (Table 1). Microglial and astrocytic responses to prion infection tended to dominate the dataset, where together they accounted for most of the increased genes (Fig. 1b).

Of the 24 genes determined to be decreased at clinical endpoint, the majority were proposed to be expressed predominantly by astrocytes, neurons, and oligodendrocytes (Fig. 1b). Some of these genes, such as *Egr1, Gnal, Hmgb1, Mal,* and *Cyrano (1700020I14Rik)*, were also significantly decreased at either 80 and/or 100 dpi (Additional File, Supplementary Dataset 2). Thus, not only does prion disease promote an increased expression of microglial- and astroglial-associated genes, it also involves the sustained reduction of several genes expressed by oligodendrocytes and neurons in the brain.

Over 300 GO: Biological Processes were determined to be statistically enriched and potentially affected during prion disease, but most of the altered genes enriched at clinical endpoint were associated with multiple immune processes, apoptosis, and phagocytosis (Fig. 2). Many of these processes are typically attributed to microglia, but other processes associated with other cells types were also evident in the dataset (Fig. 1b and Additional File, Supplementary Dataset 3). Also enriched were genes impacting neurons such as those involved in Positive regulation of neuron apoptotic process (*Ccl3, Ctsz, Egr1, Hrk, Jun, Ncf2, and Tnfrsf1a*), Negative regulation of neuron projection development (*Apoe, B2m, Ctsz, Gfap, H2-D1, H2-K1*, and *Vim*), Negative regulation of neuron apoptotic process (*Apoe, Axl, Ccl12, Hmox1, Jun,* and *Mt1*), Neuron projection morphogenesis (*Clu, Nckap1l, Rac2*, and *Thbs4*), and Positive regulation of neuron death (*Clu, Egr1, Fcgr2b*, and *Itgam*) (Additional File, Supplementary Dataset 3).

Furthermore, alterations in expression of *Hexa, Hexb, Mal*, and *Tgfb1* during prion infection (Additional File, Supplementary Dataset 3) indicated that the GO: Biological Process of Myelination was dysregulated at clinical endpoint. We could detect a decrease in *Mal* (Myelin and Lymphocyte Protein) expression at 80 and 157 dpi during prion infection. Mal is exclusively produced by oligodendrocytes in the healthy brain [29, 37] and is likely involved in myelin biogenesis and/or myelin function [38, 39]. This implied that not only are neurons damaged during prion disease, but that oligodendrocytes might be as well.

### Upregulated genes predominantly expressed by microglia during prion infection

**Fig. 3:**
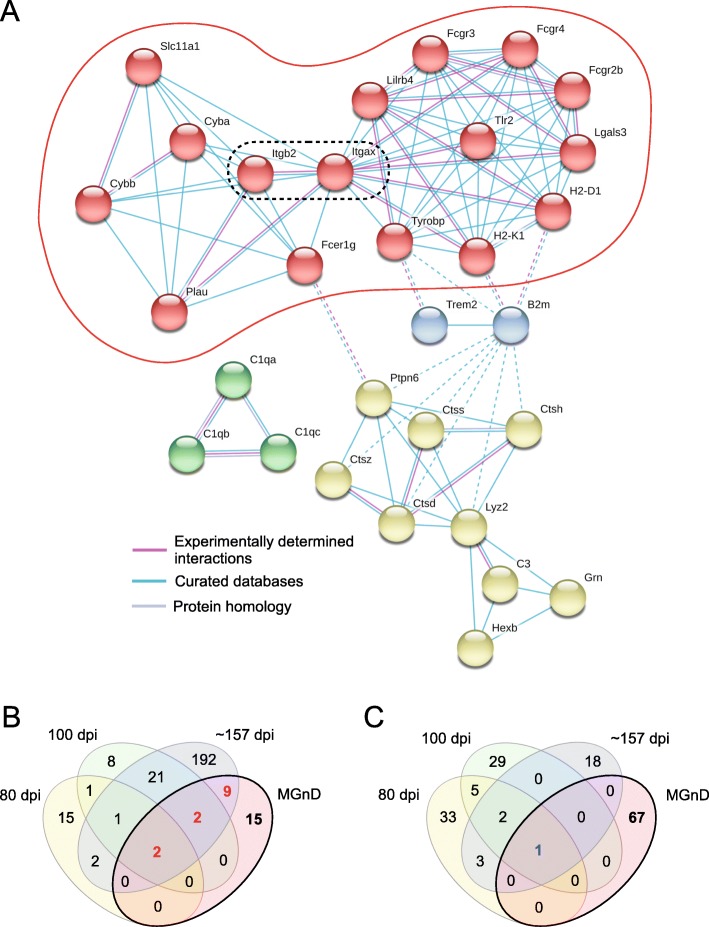
STRING network analysis of the top 57 genes predominantly expressed by microglia that are significantly increased at ≥3.0-fold in the brain during prion disease (**a**). Markov clustering indicated 4 groups of interacting protein networks. The largest group indicated by red nodes (red oval) centers around the hetero-dimeric integrin receptor CD11c/18, which is encoded by *Itgax* and *Itgb2* (black dotted oval). Experimentally determined interactions are indicated by pink lines, interactions that are curated in databases (Reactome, KEGG, etc.) are indicated by blue lines, and proteins that are homologues are indicated by purple lines. Solid lines indicate interactions within clusters, and dotted lines indicate interactions between clusters. Only connected nodes are shown, with disconnected nodes comprising 27 genes removed from view. The upregulated genes used for this analysis are found in Table 2 and Additional File, Supplementary Dataset 2. Venn diagrams comparing significantly altered genes at different times (80, 100, and ~ 157 days) post-prion infection to the gene expression pattern of microglia associated with neurodegeneration (MGnD) (**b** and **c**). The MGnD phenotype is characterized by changes in 96 transcripts. Twenty-eight inflammatory genes are increased in the MGnD phenotype (**b**), but only 13 of these inflammatory genes are significantly increased with time during prion infection. Sixty-eight homeostatic microglial genes are decreased in the MGnD phenotype (**c**), but only one of these homeostatic microglial genes is significantly decreased during prion disease. The genes altered at 80, 100, and ~ 157 dpi achieved our criteria for significance (either ≥2-fold or ≤ − 2-fold, with a p value ≤0.05). The details of expression changes in the 96 MGnD microglia-associated transcripts during prion infection can be found in Additional File, Tables S2 and S3

Our bulk-tissue RNA seq data suggested that a subset of 154 gene that are predominantly expressed by microglia [29] were increased during prion infection (Fig. 1, Table 2 and Additional File, Supplementary Dataset 2). In order to identify microglia-specific molecular functions that could be involved during infection, the top 57 genes that were statistically increased ≥3.0-fold in prion-infected mice were analyzed using the STRING network database. STRING analysis with Markov clustering revealed 4 prominent groups, and the largest group was aligned around the interaction of genes encoding CD11c (*Itgax*) and CD18 (*Itgb2*), which comprise the hetero-dimeric integrin receptor CD11c/18 (α_x_β_2_, complement receptor 4) (Fig. 3a). Ligand engagement of CD11c/18 can lead to phagocytosis, respiratory burst, and exocytosis of granular content in myeloid cells [40, 41]. Thus, it is not surprising that the two largest groups demonstrating inter-cluster interactions (Fig. 3a, red and yellow nodes) are fundamentally involved with phagocytosis, lysosomal degradation, and reactive oxygen species production.

**Table 2:** Top thirty genes predominantly expressed by microglia that are increased in the brains of prion-infected mice

Gene	Description	80 dpi^a^		100 dpi		~ 157 dpi	
		FC^b^	*P* value	FC	*P* value	FC	*P* value
*Lag3*^c^	lymphocyte-activation gene 3	**2.1**	**9.7 × 10**^**− 4**^	**4.5**	**1.2 × 10**^**− 6**^	**8.3**	**1.5 × 10**^**−9**^
*Cst7*	cystatin F	*1.5*	*2.8 × 10*^*− 2*^	**3.8**	**1.5 × 10**^**− 5**^	**20.8**	**1.5 × 10**^**− 11**^
*Clec7a*^c^	C-type lectin domain family 7, member a	*1.6*	*4.7 × 10*^*− 2*^	**2.9**	**2.6 × 10**^**− 4**^	**7.9**	**2.7 × 10**^**− 6**^
*Itgax*^c^	integrin alpha X/CD11c	1.4	6.1 × 10^− 3^	**2.8**	**1.5 × 10**^**−6**^	**9.4**	**5.1 × 10**^**− 12**^
*C1qb*	complement component 1q, beta	1.2	4.6 × 10^− 1^	**2.7**	**7.8 × 10**^**− 3**^	**4.6**	**1.5 × 10**^**− 5**^
*H2-D1*	histocompatibility 2, D region locus 1	1.3	2.5 × 10^− 2^	**2.3**	**8.3 × 10**^**− 5**^	**3.5**	**9.8 × 10**^**− 8**^
*H2-K1*	histocompatibility 2, K1, K region	1.4	1.2 × 10^− 1^	**2.3**	**2.2 × 10**^**− 4**^	**3.5**	**2.3 × 10**^**− 6**^
*B2m*	beta-2 microglobulin	1.2	4.6 × 10^− 1^	**2.2**	**9.7 × 10**^**− 4**^	**3.7**	**1.9 × 10**^**− 6**^
*Lgals3bp*	lectin, galactoside-binding, soluble, 3 binding protein	*1.7*	*3.2 × 10*^*− 2*^	**2.2**	**4.3 × 10**^**− 4**^	**4.7**	**1.2 × 10**^**− 5**^
*Fcgr4*	Fc receptor, IgG, low affinity IV	1.4	3.8 × 10^− 3^	**2.2**	**1.3 × 10**^**− 5**^	**3.4**	**1.4 × 10**^**− 7**^
*Trem2*	triggering receptor expressed on myeloid cells 2	1.3	4.5 × 10^− 2^	**2.2**	**3.6 × 10**^**− 4**^	**5.7**	**3.9 × 10**^**− 9**^
*C1qa*	complement component 1 q alpha	1.2	2.7 × 10^− 1^	**2.2**	**1.1 × 10**^**− 3**^	**5.6**	**9.5 × 10**^**− 6**^
*Fcrls*^d^	Fc receptor-like S, scavenger receptor	1.1	4.9 × 10^− 1^	**2.2**	**2.5 × 10**^**− 4**^	**4.9**	**5.4 × 10**^**− 9**^
*Bst2*	bone marrow stromal cell antigen 2	1.5	5.4 × 10^− 2^	**2.1**	**3.8 × 10**^**− 4**^	**3.6**	**1.1 × 10**^**− 5**^
*Cd52*	CD52 antigen	1.5	2.1 × 10^− 1^	**2.1**	**4.9 × 10**^**− 2**^	**3.4**	**1.1 × 10**^**− 2**^
*Ly86*	lymphocyte antigen 86	1.3	1.2 × 10^− 1^	**2.1**	**4.0 × 10**^**− 4**^	**3.7**	**1.6 × 10**^**− 7**^
*Gpnmb*^c^	glycoprotein (transmembrane) nmb	1.1	3.9 × 10^− 1^	*1.5*	*4.1 × 10*^*− 4*^	**13.6**	**1.1 × 10**^**− 11**^
*Lyz2*	lysozyme 2	0.9	6.0 × 10^− 1^	*1.6*	*2.0 × 10*^*− 2*^	**11.8**	**2.0 × 10**^**− 9**^
*C1qc*	complement component 1 q C chain	0.7	5.6 × 10^− 1^	1.1	7.7 × 10^− 1^	**5.8**	**2.8 × 10**^**− 3**^
*Lcn2*	lipocalin 2	1.0	7.1 × 10^− 1^	1.2	1.8 × 10^− 1^	**4.9**	**6.9 × 10**^**− 9**^
*Ctss*	cathepsin S	1.2	4.0 × 10^− 1^	*1.8*	*1.9 × 10*^*− 3*^	**4.8**	**8.5 × 10**^**− 8**^
*Itgb2*	integrin beta 2	1.1	1.3 × 10^− 1^	*1.8*	*5.5 × 10*^*− 5*^	**4.6**	**4.3 × 10**^**− 11**^
*Fcgr2b*	Fc receptor, IgG, low affinity Iib	1.1	3.6 × 10^− 1^	*1.8*	*3.4 × 10*^*− 4*^	**4.5**	**9.1 × 10**^**− 9**^
*Fcgr2b*	Fc receptor, IgG, low affinity Iib splicing variant	1.2	1.7 × 10^− 1^	*1.8*	*1.5 × 10*^*− 4*^	**4.5**	**4.0 × 10**^**− 9**^
*Fcer1g*	Fc receptor, IgE, high affinity I, gamma	1.2	3.7 × 10^− 1^	*1.9*	*3.4 × 10*^*− 3*^	**4.4**	**3.8 × 10**^**− 7**^
*Ly9*	lymphocyte antigen 9	1.1	1.9 × 10^− 1^	*1.7*	*7.7 × 10*^*− 4*^	**4.3**	**3.7 × 10**^**− 9**^
*Tyrobp*	TYRO protein tyrosine kinase binding protein	1.3	1.5 × 10^− 1^	*1.7*	*7.8 × 10*^*− 3*^	**4.3**	**1.3 × 10**^**− 7**^
*C3*	complement component 3	0.9	4.2 × 10^− 1^	1.2	3.3 × 10^− 2^	**4.2**	**2.2 × 10**^**− 9**^
*Cyba*	cytochrome b-245, alpha polypeptide	1.1	4.8 × 10^− 1^	*1.8*	*4.0 × 10*^*− 3*^	**4.2**	**1.3 × 10**^**− 7**^
*Ccl3*	chemokine (C-C motif) ligand 3	1.2	1.9 × 10^− 1^	*1.9*	*4.8 × 10*^*− 4*^	**4.1**	**6.7 × 10**^**− 9**^

Bolded values denote genes increased ≥2.0-fold with *p* values ≤0.05 (5.0 × 10^− 2^) in RML-infected mice

Values in italics are increased between 1.5-fold and 1.9-fold with *p* values ≤0.05 (5.0 × 10^− 2^) in RML-infected mice

^a^*dpi* days post inoculation

^b^*FC* fold change

^c^ Genes that are also increased in the MGnD phenotype

^d^ Gene that is decreased in the MGnD phenotype but increased during prion disease

### During prion infection there is no evidence of the MGnD phenotypic response associated with neurodegeneration

The MGnD microglia phenotype is defined by alterations in 96 genes in total, with the upregulation in 28 inflammatory genes and the reduction of 68 homeostatic microglial genes [14]. Assessment of prion-infected mouse transcripts increased at 80-, 100-, and ~ 157-dpi indicated that 13 of the 28 upregulated inflammatory genes associated with MGnD microglia were significantly increased during prion disease over time (Fig. 3b). Of the 68 homeostatic microglial genes that are reduced in MGnD microglia, only one gene (*Egr1*) associated was significantly decreased during prion disease (Fig. 3c). We also noted that 5 of these homeostatic genes typically decreased in MGnD microglia (*Csf1r*, *Fcrls, Hexb*, *Jun* and *Tgfb1*) were increased in prion disease over time, with two (*Fcrls* and *Jun*) upregulated at 100 dpi. Based on our bulk RNA-seq analysis of the infected CNS, we infer that during prion disease there is no evidence to support a MGnD phenotypic response. Details of these alterations in MGnD-associated microglia genes during prion disease can be found in Additional Files, Tables S2 and S3.

**Fig. 4:**
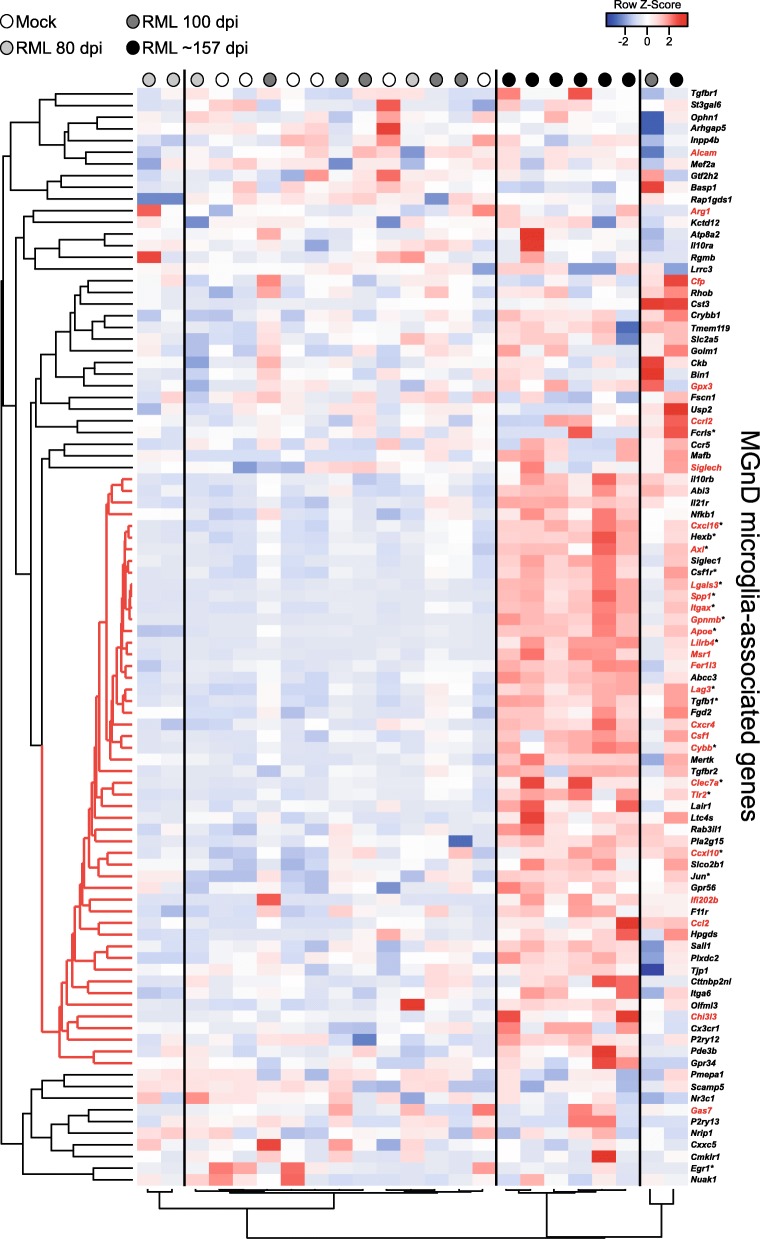
Pearson correlation heatmap and average linkage hierarchical cluster analysis of the row Z-scores obtained from our RNA-seq dataset from genes associated with the MGnD microglia phenotype. The gene designations are to the right of the heatmap. Genes in black are decreased in the MGnD microglia. Genes in red are increased in MGnD microglia. The mouse groups are color coded (refer to key on the figure) for ease of comparison. A core cluster representing a mix of 51 genes that are typically increased or decreased in MGnD microglia, which display a coordinated pattern of expression during prion disease, are indicated with red distance lines to the left of the dendrogram. An asterisk next to the gene name indicates these genes are significantly different in our RNA-seq analysis for prion-infected mice relative to mock-infected mice

A hierarchical cluster analysis of the altered 96 genes associated with the MGnD phenotype from our RNA-seq analysis further demonstrates the differences between the transcript levels during prion disease and the MGnD phenotype (Fig. 4). The mice cluster into 2 major clades with prion-infected mice at 80 dpi (light grey dots) and 100 dpi (dark grey dots) largely indistinguishable from mock-infected mice (white dots). Mice that are at the clinical endpoint (~ 157 dpi, black dots) cluster tightly, and the overall analysis indicates a distinct subset of 51 genes that show a similar pattern of expression (Fig. 4, red distance lines on the left dendrogram). This distinct gene cluster includes several MGnD microglial genes defined as homeostatic and inflammatory that are significantly increased in the brain during prion infection (Fig. 4, asterisked genes). Thus, the expression pattern of microglial genes in the prion-infected brain differs from that found in other models of neurodegenerative disease.

### RNA-seq comparison of prion-infected mice with microglia ablated (PLX5622 treated) versus infected untreated mice

Our RNA-seq analysis of prion-infected mice at mid-incubation, preclinical, and clinical times agreed with previous studies indicating that the microglial response is extensive during prion disease [35]. Our previous findings, where microglia were ablated by treatment with CSF-1R inhibitor PLX5622, established that microglia are beneficial and critical to host defense when mice are challenged with prions [13]. We were curious as to the mechanisms that might be involved in the acceleration of disease in the absence of microglial influences.

Initially we assessed if PLX5622 treatment alone was influencing gene expression and compared uninfected mice that were either PLX5622-treated or untreated (Additional File, Supplementary Dataset 4). As expected, the majority (~ 71%) of the genes significantly reduced with PLX5622 treatment were microglia-associated (i.e. *Csf1r*, *Ctss*, *Cx3cr1*, *C1qa*, *P2ry12*, etc.). This further supported our original assessment that microglia were ablated with treatment [13]. The remaining 31 genes not associated with microglia that were influenced by PLX5622-treatement alone, though statistically altered based on our criteria, had q values between 0.19 to 0.87, indicating a high probability that these might be false changes. Furthermore, none of these 37 genes were altered in our future comparisons, except for gene *Tmem261*. *Tmem261* was found to be equally decreased (~ 2-fold) in all comparisons of PLX5622-treated and untreated mice and might be altered due to PLX5622 treatment alone.

With the confidence that ablation of microglia was supported by our RNA-seq analysis and the influence of drug alone on additional transcripts was minimal, we performed RNA-seq analysis on the brains of PLX5622-treated mice infected with RML prions at 80 dpi, 100 dpi, and clinical endpoint (an average of ~ 127 dpi) and compared the result to the RNA-seq data acquired from similarly infected mice that were untreated (Additional File, Table S1 and Additional File, Supplementary Dataset 5). GO enrichment analysis of the differentially expressed genes was performed to understand what accompanying Biological Processes, Cellular Components, and Molecular Functions are dysregulated in mice that are ablated of microglia and succumb to prion disease earlier (Additional File, Supplementary Dataset 6).

**Fig. 5:**
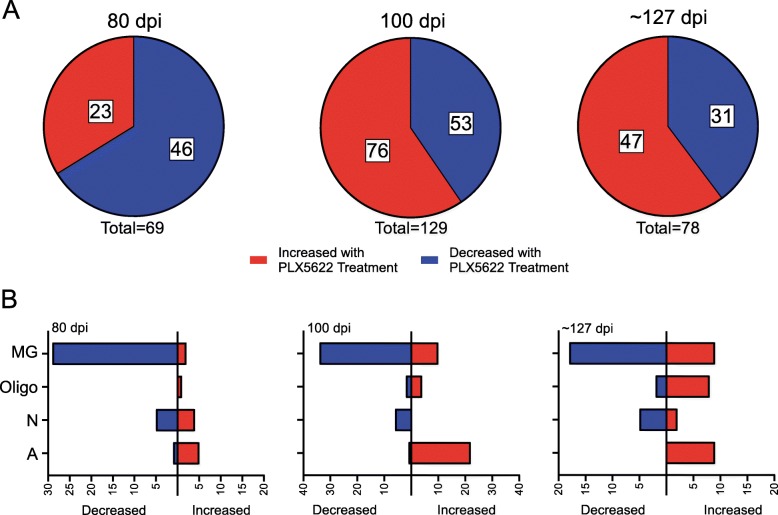
Summary of differentially expressed genes resulting from RNA-seq comparisons of RML-infected PLX5622-treated mice at 80 dpi,100 dpi, and clinical endpoint (~ 127 dpi) relative to RML-infected Untreated mice at 80 dpi, 100 dpi, and clinical endpoint (~ 157 dpi). In panel **a**, the red indicates the proportion of genes increased in the brains of PLX5622-treated mice, while blue represents the proportion of genes decreased in the brains of PLX5622-treated mice. Panel **b** indicates the number of differentially expressed genes either increased (red bars) or decreased (blue bars) that are predominately expressed in the indicated cells of the CNS during basal conditions. MG refers to microglia, Oligo refers to oligodendrocytes, N refers to neurons, and A refers to astrocytes. Genes that are similarly expressed in more than one cell type are not shown for simplicity. The specific genes, their fold change, their cellular expression, and statistical analysis can be found in Additional File, Supplementary Dataset 5

**Fig. 6:**
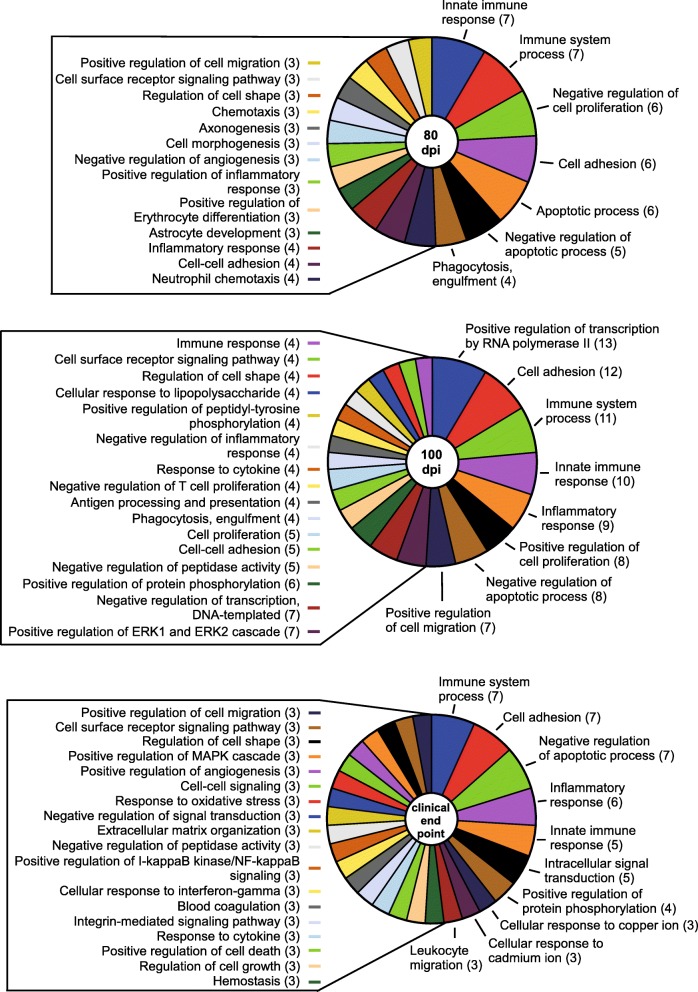
Itemization of the GO: Biological Processes that were statistically enriched when comparing PLX5622-treated RML-infected mice to Untreated RML-infected mice by RNA-seq at 80, 100, and clinical endpoint (~ 127 for PLX5622-treated and ~ 157 for Untreated). The affected categories are indicated with the number of genes that were differential expressed that are associated with that specific biological process in parentheses. Not all enriched biological processes could be included for all time points but can be found in Additional File, Supplementary Dataset 6, where a full analysis of GO: Biological Processes, Cellular Component, and Molecular Function and the associated genes can be found

At 80 dpi PLX5622-treated mice averaged a 91% reduction in microglia (Additional File, Table S1), and there were 46 genes significantly decreased in PLX5622-treated relative to untreated mice (Fig. 5a and Additional File, Supplementary Dataset 5). Predictably, most of the genes decreased in PLX5622-treated mice were predominantly expressed by microglial cells (~ 64%) and included *Cx3cr1*, *Csf1r*, and *P2ry12* (Fig. 5b). Again, we focused our analysis on the GO category of Biological Processes, where we determined that alterations in 33 Biological Processes were statistically enriched when RML infected PLX5622-treated and untreated mice were compared (Fig. 6). Several GO: Biological Processes associated with various aspects of the Immune system, Apoptotic process, and Phagocytosis were adversely affected with PLX5622 treatment (Fig. 6). Also decreased were *Ndn*, *Arf3*, and *Olfm3* that are mainly expressed in neurons (Additional File, Supplementary Dataset 5). *Ndn* was identified in our previous RNA-seq analysis above and was only detectable in untreated prion-infected mice at 80 dpi. *Arf3* encodes for ADP Ribosylation Factor 3, a small guanine nucleotide-binding protein involved in vesicular trafficking [42, 43]. *Olfm3* encodes for Olfactomedin 3, which is a member of a group of proteins that might regulate axonal growth and are involved with normal responses to olfactory stimuli [44].

Twenty-three genes were increased at 80 dpi in PLX5622-treated relative to untreated mice (Fig. 5a and Additional File, Supplementary Dataset 5). Notable were genes associated with Axonogenesis (*Lrrn3* and *Slitrk5*) and Astrocyte development (*Gfap*, *S100a8*, and *Vim*) (Additional File, Supplementary Dataset 6). Interestingly, several markers expressed by Pan-reactive astrocytes (*Vim, Serpina3n*, and *Gfap*) were increased (Table 3). This suggested that astrocytes were more activated at 80 dpi when microglia were ablated.

**Table 3:** Analysis of Pan, A1, and A2 astrocyte signature gene expression by RNA-seq comparing prion-infected PLX5622-treated mice (microglia ablated) to similarly infected untreated mice (microglia present)

Gene	Description	80 dpi^a^		100 dpi		Terminal^c^	
		FC^b^	*P* value	FC	*P* value	FC	*P* value
Pan							
*Aspg*	Asparaginase	*1.8*	*3.0 × 10*^*− 3*^	**2.6**	**1.2 × 10**^**− 6**^	*1.5*	*1.9 × 10*^*− 5*^
*Cd44*	CD44 antigen	1.6	5.9 × 10^− 2^	**4.2**	**6.7 × 10**^**− 6**^	**2.0**	**3.2 × 10**^**− 5**^
*Gfap*	Glial fibrillary acidic protein	**4.2**	**6.8 × 10**^**− 4**^	**4.5**	**1.7 × 10**^**− 7**^	*1.7*	*4.1 × 10*^*− 6*^
*Gfap*	Splice variant id MSTRG.4106	*1.9*	*2.9 × 10*^*− 2*^	**3.2**	**1.5 × 10**^**− 6**^	*1.9*	*1.1 × 10*^*− 4*^
*Gfap*	Splice variant id MSTRG.4107	1.1	4.7 × 10^− 1^	*1.8*	*4.8 × 10*^*− 3*^	*1.5*	*3.8 × 10*^*− 3*^
*Hspb1*	Heat shock protein 1	1.6	1.2 × 10^− 1^	**2.2**	**4.8 × 10**^**− 2**^	**2.1**	**2.7 × 10**^**− 2**^
*Lcn2*	Lipocalin 2	1.5	1.4 × 10^− 1^	**2.1**	**8.9 × 10**^**− 3**^	**2.1**	**3.9 × 10**^**− 3**^
*Osmr*	Oncostatin M receptor	*1.8*	*3.0 × 10*^*− 2*^	**4.2**	**1.1 × 10**^**− 5**^	**2.1**	**3.6 × 10**^**− 4**^
*Serpina3n/3f/3 m*	Serine (or cysteine) peptidase inhibitor, clade A, member 3N /3F/ 3M	**3.5**	**4.1 × 10**^**− 4**^	**5.0**	**1.1 × 10**^**− 5**^	**2.0**	**1.1 × 10**^**− 4**^
*Timp1*	Tissue inhibitor of metalloproteinase 1	1.5	1.3 × 10^− 1^	**2.2**	**1.6 × 10**^**− 5**^	*1.8*	*1.3 × 10*^*− 4*^
*Vim*	Vimentin	**2.6**	**9.8 × 10**^**− 3**^	**5.7**	**1.2 × 10**^**− 4**^	**3.8**	**1.1 × 10**^**− 6**^
A1							
*A2m*	Alpha-2-macroglobulin	1.4	8.5 × 10^− 2^	**3.4**	**6.3 × 10**^**− 5**^	*1.9*	*3.3 × 10*^*− 6*^
*C3*	Complement component 3	1.1	2.8 × 10^− 1^	**2.4**	**6.6 × 10**^**− 3**^	*1.7*	*3.5 × 10*^*− 5*^
*C4b*	Complement component 4B (Chido blood group)	**2.9**	**1.6 × 10**^**− 4**^	**3.0**	**1.2 × 10**^**− 6**^	*1.5*	*4.7 × 10*^*− 6*^
*Endou*	Endonuclease, polyU-specific	1.3	2.8 × 10^− 2^	*1.5*	*4.3 × 10*^*− 5*^	1.2	2.1 × 10^− 2^
*Fbln5*	Fibulin 5	1.1	2.6 × 10^− 2^	1.2	4.0 × 10^− 2^	*1.9*	*4.7 × 10*^*− 4*^
*Fkbp5*	FK506 binding protein 5	0.7	6.4 × 10^− 2^	1.0	8.3 × 10^− 1^	1.6	6.5 × 10^− 2^
*Ggta1*	Glycoprotein galactosyltransferase alpha 1, 3	*1.5*	*3.1 × 10*^*− 2*^	**2.4**	**3.2 × 10**^**− 5**^	1.4	1.7 × 10^− 1^
*Serping1*	Serine (or cysteine) peptidase inhibitor, clade G, member 1	1.6	7.8 × 10^− 2^	*1.6*	*1.9 × 10*^*− 2*^	**2.0**	**1.6 × 10**^**− 5**^
*Slc22a4*	Solute carrier family 22 (organic cation transporter), member 4	1.3	3.3 × 10^− 3^	*1.5*	*7.3 × 10*^*− 6*^	*1.5*	*3.1 × 10*^*− 4*^
*Slc39a14*	Solute carrier family 39 (zinc transporter), member 14	0.9	7.2 × 10^− 1^	*1.5*	*5.0 × 10*^*− 2*^	1.2	1.8 × 10^− 1^
*Sorbs1*	Sorbin and SH3 domain containing 1	1.0	9.7 × 10^− 1^	*1.5*	*4.0 × 10*^*− 3*^	1.3	1.1 × 10^− 1^
*Sulf2*	Sulfatase 2	1.0	8.6 × 10^− 1^	1.4	6.3 × 10^− 3^	*1.7*	*7.2 × 10*^*− 7*^
*Tspan4*	Tetraspanin 4	1.2	4.6 × 10^− 1^	1.3	1.6 × 10^− 2^	*1.5*	*5.1 × 10*^*− 2*^
A2							
*Anxa2*	Annexin A2	1.3	2.8 × 10^− 1^	*1.6*	*4.9 × 10*^*− 3*^	*1.6*	*1.4 × 10*^*− 4*^
*Cd109*	CD109 antigen	1.2	2.1 × 10^− 1^	**2.6**	**3.8 × 10**^**− 4**^	**2.7**	**4.3 × 10**^**− 8**^
*Emp1*	Epithelial membrane protein 1	1.2	9.1 × 10^− 2^	**2.2**	**2.6 × 10**^**− 4**^	1.5	7.4 × 10^− 2^
*Flnc*	Filamin C, gamma	*1.5*	*1.7 × 10*^*− 2*^	**2.9**	**1.0 × 10**^**− 5**^	**2.1**	**1.7 × 10**^**− 7**^
*Gadd45b*	Growth arrest and DNA-damage-inducible 45 beta	1.2	5.3 × 10^− 1^	*1.5*	*6.7 × 10*^*− 4*^	1.4	5.8 × 10^− 2^
*Hmox1*	Heme oxygenase 1	*1.5*	*6.8 × 10*^*− 3*^	*1.7*	*6.2 × 10*^*− 4*^	1.2	2.0 × 10^− 2^
*Lgals3*	Lectin, galactose binding, soluble 3	1.3	5.4 × 10^− 2^	*1.6*	*1.3 × 10*^*− 3*^	1.5	7.8 × 10^− 2^
*Lif*	Leukemia inhibitory factor	1.1	5.8 × 10^− 2^	*1.7*	*6.3 × 10*^*− 5*^	1.3	8.4 × 10^− 5^
*Ptx3*	Pentraxin related gene	1.2	1.2 × 10^−1^	**2.0**	**8.2 × 10**^**− 4**^	*1.5*	*8.4 × 10*^*− 5*^
*S100a10*	S100 calcium binding protein A10 (calpactin)	1.8	1.5 × 10^−1^	**2.3**	**9.7 × 10**^**− 3**^	1.5	9.3 × 10^− 2^
*Sbno2*	Strawberry notch homolog 2	1.3	1.3 × 10^− 2^	**2.0**	**9.5 × 10**^**− 6**^	1.4	2.5 × 10^− 6^
*S1pr3*	Sphingosine-1-phosphate receptor 3	1.5	3.9 × 10^−1^	**4.1**	**3.0 × 10**^**− 6**^	1.0	9.8 × 10^− 1^
*Tgm1*	Transglutaminase 1, K polypeptide	*1.5*	*6.8 × 10*^*− 3*^	**3.4**	**4.7 × 10**^**− 5**^	*1.6*	*3.0 × 10*^*− 4*^
*Tm4sf1*	Transmembrane 4 superfamily member 1	1.1	3.2 × 10^−1^	*1.7*	*1.8 × 10*^*− 3*^	*1.7*	*2.1 × 10*^*− 5*^
*Tnfrsf12a*	Tumor necrosis factor receptor superfamily, member 12a	1.3	2.3 × 10^−1^	**2.1**	**5.3 × 10**^**−3**^	1.8	3.1 × 10^− 3^

Bolded values denote genes increased ≥2.0-fold with *p* values ≤0.05 (5.0 × 10^− 2^) in RML-infected mice

Values in italics are increased between 1.5-fold and 1.9-fold with *p* values ≤0.05 (5.0 × 10^− 2^) in RML-infected mice

^a^*dpi* days post inoculation

^b^*FC* fold change

^c^ Terminal comparison is RML-infected mice at ~ 157 dpi relative to PLX5622-treated RML-infected mice at ~ 127 dpi

RML-infected mice treated with PLX5622 averaged a 61% reduction in microglia relative to control mice (Additional File, Table S1). Comparing PLX5622-treated and untreated datasets at 100 days after prion infection, we identified 129 genes that met our criteria as differently expressed (Fig. 5a and Additional File, Supplementary Dataset 5). Of the 60 GO: Biological Processes identified as enriched with 2 or more genes represented, many immune processes (Innate immune response, Inflammatory response, Phagocytosis, Antigen processing, Response to cytokine, etc.) were altered with PLX5622 treatment (Fig. 6). This was not unexpected with the reduction of microglia in the brains of treated mice [13]. Other notable GO processes that were influenced by PLX5622 treatment were the enrichment of genes involved in the Negative regulation of neuron projection development (*Gfap*, *Lgals1*, and *Vim*) and the Axon cellular component (*C4a, C4b, Hspb1, Kcna1, Syt2*, and *Vim*) (Additional File, Supplementary Dataset 6). Thus, during prion infection with microglia ablation there was evidence of astrocyte (*Gfap, Vim, C4a*, and *C4b*), oligodendrocyte (*Kcna1*, *S100a6*, and *Klk6*), and neuronal (*Syt2*, *Tbr1*, and *Ppp3r1*) gene dysregulation exerting a possible negative effect on the CNS.

When we compared prion-infected mice treated with PLX5622 to prion-infected untreated mice at the clinical endpoint (~ 127 to ~ 157, respectively), we identified 78 genes that were differentially expressed (Fig. 5a and Additional File, Supplementary Dataset 5). Though we have previously shown that microglial expansion occurs in PLX5622-treated mice late in the disease process [13] (Additional File, Table S1), there was evidence of decreased transcription of genes predominantly expressed by microglia (Fig. 5b) and disruption of multiple immune system processes when comparing RNA-seq datasets from clinical PLX5622-treated to untreated mice (Fig. 6). Furthermore, genes involved in Negative regulation of apoptotic process (*Cd74, Cdkn1a, Csf1r, Cyr61, Mmp9, Prnp,* and *Socs3*), Regulation of cell death (*Cdkn1a, Prnp,* and *Trim13*), and Response to oxidative stress (*Mmp9, Prdx6*, and *Prnp*) were enriched by GO analysis, with many of these genes increased in the PLX5622-treated group (Fig. 6, Additional File, Supplementary Datasets 5 and 6). Interestingly, *Prnp* was increased approximately 3-fold in PLX5622-treated mice (Additional File, Supplementary Dataset 5). *Prnp* encodes for the cellular prion protein that is the substrate converted into the infectious, misfolded isoform PrPSc [4]. Thus, dysregulation of these processes may contribute to the acceleration of prion disease in PLX5622-treated mice [13].

### Assessment of Pan-, A1-, A2- reactive astrocyte markers during prion disease

**Fig. 7:**
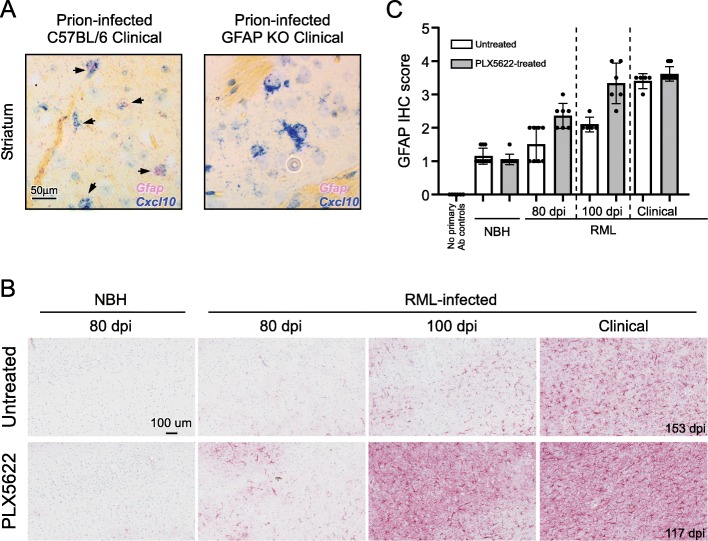
Representative dual colorimetric in situ hybridization (CISH) to determine cellular population expressing *Cxcl10* (**a**). Prion-infected C57BL/6 and GFAP-deficient (GFAP KO) mice were inoculated with prions and allowed to advance to clinical disease. Sections from formalin fixed brains were hybridized with probes specific for *Cxcl10* (blue) or *Gfap* (pinkish-red). Infected C57BL/6 mice had several large nuclei demonstrating expression of both *Gfap* and *Cxcl10* (arrows) within the cell in several regions including the cerebral cortex (not shown), cerebellum, (not shown) and striatum (shown). GFAP KO mice displayed many cells in these same regions that expressed *Cxcl10* that lacked hybridization with the *Gfap* probe, demonstrating the hybridization with *Gfap* was specific. The scale for both representative images is indicated in the first image in A. Representative immunohistochemical assessment of astrogliosis in cerebral cortex brain sections from Untreated and PLX5622-treated mice (**b**). Mice were inoculated with either normal brain homogenate (NBH) or scrapie strain RML. Sections of cerebral cortex from mice at the dpi indicated were probed with antibodies against GFAP. Representative images of the cerebral cortex are shown for all at the scale indicated in the first panel. The dpi of the clinical mice is as indicated. The intensity of GFAP histopathological immunoreactivity observed from stained coronal sections (Additional File, Figure S1) was given a subjective score from 0 to 4 based on the amount and distribution of GFAP staining in the brain (see methods) and is graphically depicted (**c**). No primary antibody (Ab) controls were absent of staining and defined as 0

Our initial RNA-seq comparison of RML prion-infected to uninfected mice at mid-incubation (80 dpi) suggested one of the first indicators of disease was an increase in the expression of *Cxcl10* (Table 1). *Cxcl10* is associated with astrocytes as a Pan-reactive marker and also with microglia that assume the MGnD phenotype*.* We performed dual colorimetric in situ hybridization (CISH) using probes to *Gfap* (fast-red) and *Cxcl10* (fast-blue) on fixed brain sections from prion-infected C57BL/6 mice and GFAP-deficient mice as controls (Fig. 7a). Our CISH results suggested that during prion disease, the predominant cell type producing *Cxcl10* was astrocytes. The increase in *Cxcl10* by astrocytes was followed by upregulation of additional Pan-reactive genes (*Gfap* and *Serpina3n*) at 100 dpi along with increased expression of *B2m, C4b, H2-D1* and *Ifitm3/6*, genes associated with A1-reactive astrocytes. As prion disease progressed from the early stages to clinical disease, it was evident that additional Pan-, A1-, and A2- associated genes were increased with time (Table 1).

Microglia are thought to be responsible for inducing the A1 phenotype in astrocytes under pro-inflammatory conditions [16]; therefore, the influence of microglia on inducing reactive astrocytes during prion disease was assessed using PLX5622-treated mice. At 80 dpi the Pan-reactive astrocyte genes *Gfap, Serpina3n,* and *Vim* were increased when microglia were ablated (Table 3), but the increase in *Cxcl10* expression was comparable to untreated prion-infected mice (Additional File, Supplementary Datasets 7 and 8, compare FPKM values for *Cxcl10*). Ultimately, microglia ablation with PLX5622 treatment did not reduce *Cxcl10* expression at any time analyzed. This further supported our CISH results and indicated that the increase in *Cxcl10* expression at 80 dpi was associated with the presence of Pan-reactive astrocytes in the brain. Furthermore, the expression of A1-associated gene *C4b* was also elevated in PLX5622-treated mice during prion disease at this early time point (Table 3).

RNA-seq comparison of PLX5622-treated and untreated datasets at 100 dpi also indicated additional Pan-, A1-, and A2-reactive astrocyte genes were more highly expressed when microglia were ablated (Table 3). We could detect increases in 8 Pan-reactive genes (*Vim, Serpina3n, Gfap, Cd44, Aspg, Timp1, Hspb1*, and *Lcn2*), as well as increased expression of A1-associated genes (*A2m*, *C4b, Ggta1*, and *C3*) and A2-associated genes (*Tgm1, Flnc, Cd109, A100a10, Emp1, Tnfrsf12a, Sbno2*, *S1pr3,* and *Ptx3*). At the clinical endpoint, we noted fewer differences in the expression of Pan-, A1-, and A2-reactive astrocyte phenotypic genes between the groups. Thus, astrocytes are more similarly activated at the end stage of prion disease regardless of PLX5622 treatment.

RNA-seq analysis indicated that transcription of Pan-reactive astrocyte marker *Gfap* was increased in the brains of prion-infected mice when microglia were abated by PLX5622 treatment. We further assessed GFAP and astrogliosis in the brain by comparing prion-infected PLX5622-treated and untreated mice by immunohistochemistry (IHC) (Fig. 7b and c). GFAP immunostaining was greater in PLX5622-treated mice compared untreated mice at 80 and 100 dpi but less evident at the clinical endpoint. The distribution and pattern of immunostaining indicated the increase in GFAP began as regional foci first in the thalamus and secondarily in the cortex (Additional File, Figure S1), which matched our previous findings of the deposition of PrPSc in the brain [45]. The distribution of GFAP immunostaining eventually encompassed almost the entire brain at clinical endpoint regardless of treatment. Thus, the staining of GFAP suggested that astrogliosis in the brains of prion-infected mice was accelerated when microglia were ablated but followed a similar progression as untreated mice.

**Fig. 8:**
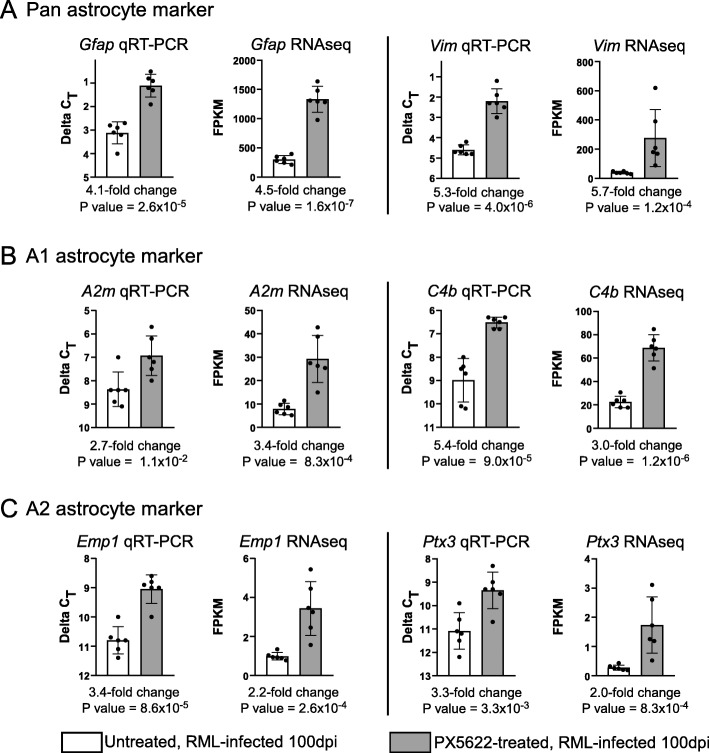
Validation of a selection of altered Pan- (panel **a**), A1- (panel **b**), and A2- (panel **c**) reactive astrocyte associated genes altered in the brains of 100 dpi RML-infected mice by qRT-PCR. Mice that were treated with PLX5622 are grey columns and Untreated are white columns. The qRT-PCR results are presented as the Delta C_T_ values. Also present is the RNA-seq data in Fragments Per Kilobase Million (FPKM) for each gene for comparison. Each dot represents the analysis of an individual mouse. The bars represent 1 standard deviation from the mean. *P* values and fold change of PXL5622 treatment relative to untreated are below each graph. Validation results from a more comprehensive list of Pan-, A1-, and A2-reactive astrocyte markers can be found in Additional File, Figure S2

Because the 100-dpi time displayed the most differences between PLX5622-treated and untreated mice, we used these RNA samples to confirm by qRT-PCR the alterations in expression using primer sets specific to 29 reactive astrocyte-associated genes. A subset of our findings is presented in Fig. 8. The RNA-seq and qRT-PCR results were largely in agreement (Additional File, Figure S2), offering further support that during prion disease astrocyte activation in the microglia-ablated brain is intensified relative to untreated infected mice.

### Hierarchical cluster analysis of Pan-, A1-, and A2-associated gene expression during prion infection

**Fig. 9:**
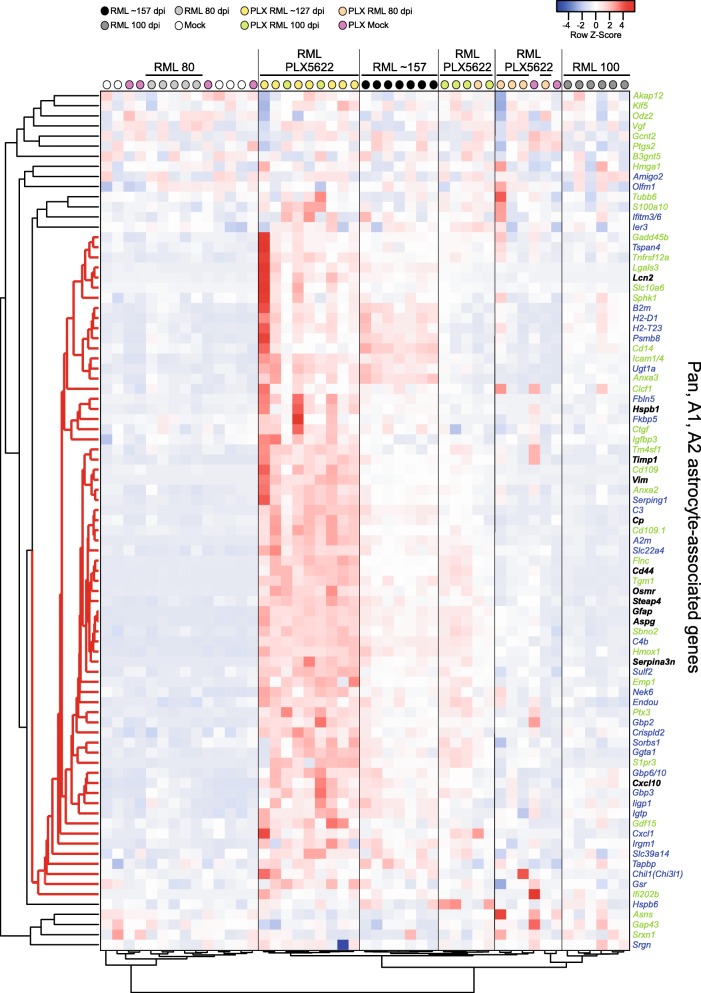
Pearson correlation heatmap and average linkage hierarchical cluster analysis of the row Z-scores obtained from our RNA-seq dataset from reactive astrocyte genes. The gene designations are to the right of the heatmap. Genes in black are associated with Pan-reactive astrocytes. Genes in blue are associated with A1-reactive astrocytes. Genes in green are associated with A2-reactive astrocytes. The mouse groups are color coded (refer to key on the figure) and several are labeled for ease of comparison. A core cluster of 66 genes representing a unique mix of Pan-, A1-, and A2-associated genes that display a coordinated pattern of expression during prion disease are indicated with red distance lines to the left of the dendrogram

An overall assessment of astrogliosis through a hierarchical cluster analysis of 85 Pan-, A1-, and A2-associated genes [16, 18] is presented as a dendrogram and heatmap in Fig. 9. Untreated RML-infected mice at 80 dpi (light grey dots) cluster with mock inoculated mice, demonstrating that astrocytes are not sufficiently activated with RML infection at this early time. Untreated RML-infected mice at 100 dpi (dark grey dots) cluster tightly, and the astrocyte gene expression is more distant relative to the other prion-infected groups. Many of the PLX5622-treated mice at 80 dpi (orange dots) cluster together, but also grouped in this cluster were 2 of the mock inoculated PLX5622-treated mice had been treated for 161 days. Most of the PLX5622-treated mice at 100 dpi (green dots) cluster tightly, with their astrocyte expression signature more closely related to clinical untreated mice at ~ 157 dpi (black dots). The most highly expressed mix of Pan-, A1- and A2-associated reactive astrocyte transcripts was observed in the PLX5622-treated mice at ~ 127 dpi (the clinical endpoint, yellow dots). These data reaffirm that during prion disease, astrocyte activation is more advanced with PLX5622 treatment. Moreover, our heatmap analysis shows a distinct expression signature of 66 genes associated with reactive astrocytes (Fig. 9, red distance lines on the left dendrogram), with an increase in expression of most of these genes when microglia are ablated.

### Canonical microglial responses are not required to induce genes associated with A1-astrocytes during prion disease

When microglia are exposed to LPS, they increase the expression and secretion of many immune effectors [46, 47]. LPS-stimulated microglial secretion of Tnf, IL-1a, and C1q has been shown to induce the reactive A1-astrocyte phenotype, with all 3 required for full recreation of the phenotype in vitro [16]. Interestingly, our RNA-seq analysis suggested that only the subunits of C1q (*C1qa, C1qb,* and *C1qc*) were significantly increased in prion-infected mice at 100-dpi or at clinical times (~ 157 dpi) relative to uninfected control mice (Table 4).

**Table 4:** Expression changes of microglial effectors in the brain that affect the A1-reactive astrocyte phenotype in RML-infected mice relative to Uninfected mice

Gene	80 dpi^a^		100 dpi		~ 157^c^ dpi	
	FC^b^	*P* value	FC	*P* value	FC	*P* value
*Tnf*	1.08	4.0 × 10^− 2^	1.12	2.0 × 10^− 4^	1.39	6.6 × 10^− 5^
*Il1a*	1.02	6.5 × 10^− 1^	1.14	1.2 × 10^− 2^	1.45	3.5 × 10^− 5^
*C1qa*	1.22	2.7 × 10^− 1^	**2.17**	**1.1 × 10**^**− 3**^	**5.62**	**9.0 × 10**^**− 6**^
*C1qb*	1.24	4.6 × 10^− 1^	**2.71**	**7.8 × 10**^**− 3**^	**4.60**	**1.5 × 10**^**− 5**^
*C1qc*	0.71	5.6 × 10^− 1^	1.12	7.7 × 10^− 1^	**5.81**	**2.8 × 10**^**− 3**^
*Tfgb1*	1.13	1.8 × 10^− 1^	*1.56*	*1.1 × 10*^*− 3*^	**2.73**	**1.6 × 10**^**− 8**^
*Fgf2*	1.03	7.5 × 10^− 1^	0.98	8.4 × 10^− 1^	0.88	3.1 × 10^− 1^

Bolded values denote genes increased ≥2.0-fold with *p* values ≤0.05 (5.0 × 10^− 2^) in RML-infected mice relative to Uninfected mice

Values in italics are increased between 1.5-fold and 1.9-fold with *p* values ≤0.05 (5.0 × 10^− 2^ in RML-infected mice relative to Uninfected mice

^a^*dpi* days post inoculation

^b^*FC* fold change

^c^ ~ 157 is the average dpi to clinical endpoint for RML-infected mice

Ablation of microglia with PLX5622 significantly decreased expression of *C1qb* in the brain at 80 and 100 dpi, yet the average expression levels of *Tnf, IL1a, C1qa,* and *C1qc* in infected PLX5622-treated mice at 80 and 100 dpi were comparable to that of infected untreated mice (Table 5) as well as uninfected mice at similar times (Additional File, Supplementary Datasets 7 and 8, refer to FPKM values for 80 and 100 dpi). Thus, treatment with PLX5622 during prion disease did not increase the levels of A1-inducing immune effectors, even though the expression of Pan- and A1-associated genes were increased (Table 3). This implied that microglial upregulation of these effectors was not required for the induction of genes associated with Pan- and A1-reactive astrocytes during prion infection.

**Table 5:** Expression changes of microglial effectors in the brain that affect the A1-reactive astrocyte phenotype in PLX5622 treatment of RML-infected mice relative to Untreated RML-infected mice

Gene	80 dpi^a^		100 dpi		~ 127^c^ dpi	
	FC^b^	*P* value	FC	*P* value	FC	*P* value
*Tnf*	−1.05	1.4 × 10^− 1^	−1.07	1.7 × 10^− 2^	1.11	3.7 × 10^− 1^
*Il1a*	− 1.18	7.0 × 10^− 4^	− 1.17	3.7 × 10^− 3^	− 1.17	2.4 × 10^− 2^
*C1qa*	− 2.26	6.7 × 10^− 2^	*− 1.76*	*1.4 × 10*^*− 2*^	−1.37	3.1 × 10^− 1^
*C1qb*	**−2.93**	**4.6 × 10**^**− 3**^	**−2.53**	**5.5 × 10**^**− 3**^	− 1.59	8.0 × 10^− 2^
*C1qc*	−1.59	5.3 × 10^− 1^	−1.95	1.3 × 10^− 1^	−1.86	1.6 × 10^− 1^
*Tgfb1*	− 1.50	5.8 × 10^− 2^	*− 1.71*	*7.0 × 10*^*− 4*^	−1.39	1.0 × 10^− 1^
*Fgf2*	1.09	4.7 × 10^− 1^	1.37	2.9 × 10^− 3^	1.13	3.1 × 10^− 1^

Bolded values denote genes decreased ≥2.0-fold with *p* values ≤0.05 (5.0 × 10^− 2^) in infected PX5622-treated mice relative to infected untreated mice

Values in italics are decreased between 1.5-fold and 1.9-fold with *p* values ≤0.05 (5.0 × 10^− 2^) in infected PX5622-treated mice relative to infected untreated mice

^a^*dpi* days post inoculation

^b^*FC* fold change

^c^ ~ 127 dpi is the average dpi to clinical endpoint for infected PLX5622-treated mice

### Signals that revert A1 reactive astrocytes to a quiescent phenotype are not upregulated during prion infection

In cell culture, A1-induced astrocytes remain activated until they receive the appropriate signal to revert to a quiescent, non-reactive phenotype. This can be accomplished by the addition of exogenous TGFβ or FGF2 to the medium [16, 48]. By RNA-seq the expression of *Fgf2* was not significantly altered at any time under any experimental condition tested during prion infection (Tables 4 and 5). Furthermore, expression of additional *Fgf* family members remained unchanged during prion disease (Additional File, Supplementary Datasets 7 and 8).

Expression of *Tgfb1* was increased only 2.7-fold in the brains of untreated clinical mice relative to uninfected control mice (Table 4), while the expression of *Tgfb2* and *Tgfb3* were unchanged under any experimental condition or time analyzed (Additional File, Supplementary Datasets 7 and 8). PLX5622 treatment at 80 and 100 dpi led to modest reductions in expression of *Tgfb1* relative to untreated mice (Table 5 and Additional File, Supplementary Dataset 7), but the fold-change did not meet our criteria for being significantly decreased. Comprehensively, the effectors described to revert activated A1- astrocytes to a quiescent state [16] were mostly unchanged in response to prion infection, indicating that astrocyte activation in the CNS may proceed unchecked with prion infection.

## Discussion

Microglial activation is an early event during prion disease that occurs prior to neuronal loss and spongiform change in the brain during prion disease [6, 49]. During prion disease, the microglial response was so strong that it eventually dominated the changes within our RNA-seq dataset. In our study the genes encoding CD11c (*Itgax*) and CD18 (*Itgb2*) were increased during prion infection, indicating signaling through this hetero-dimeric integrin receptor may be important in microglial-host defense [13] in facilitating removal of PrPSc and damaged cells. Microglia have been shown to increase their expression of CD11c during aging and in response to tissue damage [50–53]. Furthermore, CD11c expressing microglia accumulate around amyloid plaques in mouse Alzheimer’s disease models [54, 55] and appear to be primed for phagocytosis [52]. CD11c/18 can interact with numerous ligands including iC3b, ICAM, fibrinogen, and denatured proteins [40, 56, 57], yet it is unclear what ligands are involved in CD11c/18 activation during prion infection. Targeting this integrin receptor or inducing its upregulation in microglia could facilitate more effective clearance of PrPSc, impeding the disease process.

Recently, a microglial phenotype was identified in several neurodegenerative mouse models that was dependent on signaling via the TREM2-APOE pathway [14]. These MGnD microglia could be induced through phagocytosis of apoptotic neurons or exogenous introduction of APOE into the brain. Our RNA-seq data of brain suggested that during prion disease, there was little evidence to support a MGnD microglial phenotype, rather they might acquire an alternative molecular signature with the upregulation of several microglia-specific homeostatic and inflammatory markers. Cluster analysis suggests this alternative microglial phenotype is more evident later in the disease and correlates with our previous findings that microglia are more beneficial in combating prions at later times [13]. Furthermore, deletion of *Trem2* [58] or *ApoE* [59] in mice does not affect prion pathogenesis or PrPSc accumulation, suggesting that TREM2-APOE microglia signaling described in other neurodegenerative disease models does not influence prion disease.

In contrast to previous studies using LPS stimulation [16], in the present work studying prion disease, astrocyte activation did not appear reliant on microglial induction. During prion infection, microglia appear to express a unique set of genes with little overlap with those described for other neurodegenerative diseases (Figs. 3, 4, and Table 2). Many of the homeostatic genes typically decreased in with the MGnD phenotype (Additional File, Table S3) appear to be unchanged in expression during prion infection. It is possible that our prion-associated expression signature plays a role in dampening the astrocyte response. The exact effectors involved in this intra-glial communication are unknown. One could speculate that once this prion-specific microglial signature is removed or reduced by PLX5622-treatment, astrocytes are then unchecked, becoming overactivated and expressing their own distinctive gene signature during disease (Fig. 9).

Astrocyte activation in the presence or absence of microglia might be directly contributing to the advancement of prion pathogenesis through the exacerbation of neurotoxic subtype of A1 astrocytes. Alternatively, the pronounced astrogliosis seen in PLX5622-treated mice could be a response to a faster disease tempo and damage. The described A1 and A2 reactive phenotypes might be opposite ends of the spectrum, or possibly these phenotypes are two possibilities on a continuum of multiple astrocyte activation states [15]. In this vein, the microglia-independent mixed A1/A2-reactive astrocyte expression signature described herein could represent an expression “snapshot” composed of multiple subtypes. Alternatively, the expression pattern indicated by the 66 genes highlighted in Fig. 9 could signify a unique reactive astrocyte phenotype associated with protein misfolding neurodegenerative diseases.

An increase in the expression of A1-associated genes has been observed in several neurological diseases. For example, reducing the presence of A1-reactive astrocytes has been shown to alter the disease course in mouse models of experimental autoimmune encephalomyelitis, Alzheimer’s disease, and Parkinson’s disease [15, 16, 60–62]. Recently, the influence of microglia-induced A1-reactive astrocytes during prion disease was assessed by infecting triple knockout mice lacking Tnf, IL-1a, and C1qa with prions [19]. These mice possess microglia but lack the capacity to induce A1-reactive astrocytes when injected with LPS [16]. Surprisingly, the reactive astrocytic response was largely unaltered in prion-infected triple knockout mice, but the mice died earlier than infected wildtype mice [19]. In contrast, in our study the ablation of microglia led to increased astrocyte activation during infection. Thus, microglia can influence astrocytes by suppressing overactivation during prion infection. Neuroprotection provided by microglia during prion infection in our previous publication was thought to act primarily by removing PrPSc by phagocytosis [13]. However, our current data indicates that microglia might also function by modulating populations of reactive astroglia.

Besides the activation of microglia and astroglia during prion disease, we found evidence of dysregulation in the process of myelination that is carried out by oligodendrocytes. The gene *Mal*, expressed by oligodendrocytes, was significantly decreased at several times tested during prion infection in untreated mice. Mal has been implicated in myelin biogenesis and function, influencing the formation, stabilization and maintenance of glycosphingolipid-enriched membrane microdomains [39, 63]. A reduction in Mal could lead to an impairment in oligodendrocyte trafficking of glycolipids such as GalC and sulfatides to the paranodal compartment near nodes of Ranvier [64]. In turn, reduction or loss of GalC and sulfatides in mice could lead to faulty nodes of Ranvier causing tremors, ataxia, slow nerve conductions, and early death [65]. In addition, the oligodendrocyte specific gene *Ermn,* encoding Ermin (Juxtanodin), was decreased by nearly 7-fold relative to uninfected mice at ~ 157 dpi (clinical endpoint). Ermin is involved in oligodendrocyte cytoskeletal rearrangement during myelinogenesis and node of Ranvier formation [66, 67]. Thus, the long-term decrease in *Mal* and clinical endpoint decrease of *Ermn* expression during prion infection could signify destabilized axonal-myelin sheaths and contribute to disease pathology.

In prion-infected mice, the altered expression of genes associated with apoptosis was notable. For example, genes implicated in neuronal apoptosis were enriched with or without microglial ablation. Apoptosis is one proposed mechanism of neuronal death during prion disease [68–71], but multiple pathways can lead to cellular apoptosis. During prion infection, we identified two differentially expressed transcriptional regulators associated with apoptosis. Early growth response factor-1 (*Egr1*), an immediate early gene that can mediate apoptosis through p73 [72], was decreased in all prion-infected groups. Therefore, Egr1 is unlikely to influence apoptosis or pathogenesis during prion disease. Conversely, *Jun* (c-Jun) [73, 74] was significantly increased in all prion-infected cohorts regardless of microglia ablation. We also found that *Hrk* was increased, which encodes a protein that promotes apoptosis by interacting with apoptotic inhibitors Bcl-2 and Bcl-X(L) and is downstream in the Jun-signaling cascade [75–77]. Thus, our data support previous findings that signaling events that funnel into the Jun N-terminal kinase (JNK) pathway are involved in prion pathogenesis [78, 79]. Importantly, our findings indicate that these signaling events are not dependent on the presence of microglia.

Our data imply another possible mechanism of apoptosis in neurons during prion infection. Cathepsin proteins typically reside in the lysosomal compartment of cells, but if released into the cytosol they can trigger apoptosis [80]. We identified several genes encoding cathepsins (*Ctss, Ctsd, Ctsz, Ctsh,* and *Ctsl*) that were upregulated in the prion-infected brain (Additional File, Supplementary Dataset 3), and all were similarly increased in infected PLX5622-treated mice as well (Additional File, Supplementary Dataset 8), with the exception of *Ctss* which was decreased with microglia ablation (Additional File, Supplementary Dataset 5). Lysosomal permeabilization and cathepsin-mediated neuronal apoptosis has been reported in Alzheimer’s disease [81–84] and with an in vitro prion model using SH-SY5Y human neuroblastoma cells [85]. Furthermore, lysosomes are important organelles in prion pathogenesis as potential sites for prion replication and spread in neurons [86–89], and lysosome-associated genes have been shown to be upregulated early and late during prion disease [78]. Thus, we speculate that an additional mechanism of neuronal apoptosis during prion disease might be cathepsin-mediated through lysosomal disruption.

Though we used the robust technique of RNA-seq to assess these changes, our approach is not without limitations. For example, we and others have shown that *P2ry12* is unchanged during prion disease when bulk RNA is assayed (herein and [90]), but Muth et al. found that P2ry12 protein was decreased in several regions when assayed using antibody to P2ry12 by immunohistochemistry [91]. Using bulk RNA we cannot account for regional differences that may occur in the CNS during prion infection. Furthermore, because of our use of bulk RNA from whole brain, we have at times made reasoned inferences as to the predominant cell type(s) associated with the increase in gene expression based on previous cell-specific RNA-seq studies [16, 18, 29]. Transcripts increasing in one cell type but equally decreasing in another might go unidentified. Many genes (i.e. *Cxcl10*) can be expressed by multiple cells within the CNS, and an increase in expression can shift from one cell type to another depending on the context. This can lead to difficulty in assigning expression to a cellular subset, but in the case of *Cxcl10* the use of in situ hybridization clearly indicated that astrocytes, not microglia, were the predominant expressers during prion infection of the CNS.

## Conclusions

We chronicled dysregulation of over 300 biological processes within the CNS during prion disease by RNA-seq analysis of the brains of prion infected mice. There was evidence of disruption of cellular compartments involving astrocytes, oligodendrocytes, and neurons. Furthermore, not only were immune processes in the CNS reduced with microglia ablation, but astrocyte activation was increased during prion disease. Surprisingly, canonical microglial responses were not required to induce astrocyte activation during prion infection. It is interesting that microglial ablation during prion infection increases astrocyte activation and prion disease tempo, but the relationship between astrocyte activation and prion pathogenesis remains to be determined. The recent description of the neurotoxic A1-reactive subtype of astrocyte indicates that astrocytes have the capacity to kill cells [15, 16], further research is required to determine if the mixed A1/A2-reactive astrocytes described herein also retain this capability.

## Supplementary information

**Additional file 1: Table S1.** C57BL/10 mice used throughout this study.

**Additional file 2: Table S2.** Gene expression during prion infection of the 23 transcripts typically increased in microglia associated with a neurodegenerative phenotype (MGnD).

**Additional file 3:Table S3.** Gene expression during prion infection of the 68 transcripts typically decreased in microglia associated with a neurodegenerative phenotype (MGnD).

**Additional file 4:Figure S1.** Representative immunohistochemical assessment of progressive astrogliosis in coronal sections from Untreated and PLX5622-treated mice. Mice were inoculated with either normal brain homogenate (NBH) or scrapie strain RML, and coronal sections from mice at 80 or 100 dpi were probed with antibodies against GFAP. Regions corresponding to the approximate location of the cerebral cortex (CTX) and thalamus (TH) are indicated or each. The scale for all representative images is indicated in the first panel.

**Additional file 5:Figure S2.** Expanded qRT-PCR analysis of Pan-, A1-, and A2-associated genes from RNA isolated from the brains of 100 dpi RML-infected mice. Mice that were treated with PLX5622 are grey columns and Untreated are white columns. The qRT-PCR results are presented as the Delta CT values. Also present is the RNA-seq data in Fragments Per Kilobase Million (FPKM) for each gene for comparison. Each dot represents the analysis of an individual mouse. The bars represent 1 standard deviation from the mean. *P* values and fold change of PXL5622 treatment relative to untreated are below each graph.

**Additional file 6: Dataset 1.** RNA-seq analysis statistics, gene annotation, and principal component analysis.

**Additional file 7: Dataset 2.** Mouse genes increased or decreased during prion infection at various time points relative to uninfected control mice.

**Additional file 8: Dataset 3.** Gene Ontology analysis of genes altered during prion infection at 80, 100, and ~157 dpi.

**Additional file 9: Dataset 4.** Mouse genes increased or decreased in uninfected PLX5622-treated mice relative to uninfected untreated mice.

**Additional file 10: Dataset 5.** Mouse genes increased or decreased in prion-infected PLX5622-treated mice at various time points relative to prion-infected untreated mice.

**Additional file 11: Dataset 6.** Gene Ontology analysis of genes altered during prion infection of PLX5622-treated mice at 80, 100, and ~127 dpi.

**Additional file 12: Dataset 7.** RNA-seq fragments per kilobase million (FPKM) counts for untreated mice and genes assessed in this study.

**Additional file 13: Dataset 8.** RNA-seq fragments per kilobase million (FPKM) counts for PLX5622-treated mice and genes assessed in this study.

## Data Availability

All data generated or analyzed during this study are included in this published article and its supplementary information files.

## Abbreviations

CSF-1R: Colony-stimulating factor-1 receptor
CNS: Central nervous system
PrPres or PrPSc: Protease-resistant prion protein
MGnD: Neurodegenerative microglia
LPS: Lipopolysaccharide
NBH: Normal brain homogenate
FPKM: Fragments per kilobase of transcript per million mapped reads
GO: Gene Ontology
NBF: Neutral buffered formalin

## References

[1] Walker LC, Jucker M (2015). Neurodegenerative diseases: expanding the prion concept. Annu Rev Neurosci.

[2] Caughey B, Lansbury PT (2003). Protofibrils, pores, fibrils, and neurodegeneration: separating the responsible protein aggregates from the innocent bystanders. Annu Rev Neurosci.

[3] Kraus A, Groveman BR, Caughey B (2013). Prions and the potential transmissibility of protein misfolding diseases. Annu Rev Microbiol.

[4] Prusiner SB (1991). Molecular biology of prion diseases. Science..

[5] Caughey B, Baron GS, Chesebro B, Jeffrey M (2009). Getting a grip on prions: oligomers, amyloids, and pathological membrane interactions. Annu Rev Biochem.

[6] Williams A, Lucassen PJ, Ritchie D, Bruce M (1997). PrP deposition, microglial activation, and neuronal apoptosis in murine scrapie. Exp Neurol.

[7] Booth S, Bowman C, Baumgartner R, Sorensen G, Robertson C, Coulthart M (2004). Identification of central nervous system genes involved in the host response to the scrapie agent during preclinical and clinical infection. J Gen Virol.

[8] Wong YC, Krainc D (2017). alpha-synuclein toxicity in neurodegeneration: mechanism and therapeutic strategies. Nat Med.

[9] Zilka N, Kazmerova Z, Jadhav S, Neradil P, Madari A, Obetkova D (2012). Who fans the flames of Alzheimer’s disease brains? Misfolded tau on the crossroad of neurodegenerative and inflammatory pathways. J Neuroinflammation.

[10] Zilka N, Korenova M, Novak M (2009). Misfolded tau protein and disease modifying pathways in transgenic rodent models of human tauopathies. Acta Neuropathol.

[11] Ransohoff RM (2016). How neuroinflammation contributes to neurodegeneration. Science..

[12] Amor S, Peferoen LA, Vogel DY, Breur M, van der Valk P, Baker D (2014). Inflammation in neurodegenerative diseases--an update. Immunology..

[13] Carroll JA, Race B, Williams K, Striebel J, Chesebro B (2018). Microglia are critical in host defense against prion disease. J Virol.

[14] Krasemann S, Madore C, Cialic R, Baufeld C, Calcagno N, El Fatimy R (2017). The TREM2-APOE pathway drives the transcriptional phenotype of dysfunctional microglia in neurodegenerative diseases. Immunity..

[15] Liddelow SA, Barres BA (2017). Reactive astrocytes: production, function, and therapeutic potential. Immunity..

[16] Liddelow SA, Guttenplan KA, Clarke LE, Bennett FC, Bohlen CJ, Schirmer L (2017). Neurotoxic reactive astrocytes are induced by activated microglia. Nature..

[17] Liddelow SA, Sofroniew MV (2019). Astrocytes usurp neurons as a disease focus. Nat Neurosci.

[18] Zamanian JL, Xu L, Foo LC, Nouri N, Zhou L, Giffard RG (2012). Genomic analysis of reactive astrogliosis. J Neurosci.

[19] Hartmann K, Sepulveda-Falla D, Rose IVL, Madore C, Muth C, Matschke J (2019). Complement 3(+)-astrocytes are highly abundant in prion diseases, but their abolishment led to an accelerated disease course and early dysregulation of microglia. Acta Neuropathol Commun.

[20] Elmore MR, Najafi AR, Koike MA, Dagher NN, Spangenberg EE, Rice RA (2014). Colony-stimulating factor 1 receptor signaling is necessary for microglia viability, unmasking a microglia progenitor cell in the adult brain. Neuron..

[21] Elmore MR, Lee RJ, West BL, Green KN (2015). Characterizing newly repopulated microglia in the adult mouse: impacts on animal behavior, cell morphology, and neuroinflammation. PLoS One.

[22] Dagher NN, Najafi AR, Kayala KM, Elmore MR, White TE, Medeiros R (2015). Colony-stimulating factor 1 receptor inhibition prevents microglial plaque association and improves cognition in 3xTg-AD mice. J Neuroinflammation.

[23] Martin M (2011). Cutadapt removes adapter sequences from high-throughput sequencing reads. Embnet J.

[24] Kim D, Langmead B, Salzberg SL (2015). HISAT: a fast spliced aligner with low memory requirements. Nat Methods.

[25] Pertea M, Pertea GM, Antonescu CM, Chang TC, Mendell JT, Salzberg SL (2015). StringTie enables improved reconstruction of a transcriptome from RNA-seq reads. Nat Biotechnol.

[26] Frazee AC, Pertea G, Jaffe AE, Langmead B, Salzberg SL, Leek JT (2015). Ballgown bridges the gap between transcriptome assembly and expression analysis. Nat Biotechnol.

[27] Babicki S, Arndt D, Marcu A, Liang Y, Grant JR, Maciejewski A (2016). Heatmapper: web-enabled heat mapping for all. Nucleic Acids Res.

[28] Szklarczyk D, Gable AL, Lyon D, Junge A, Wyder S, Huerta-Cepas J (2019). STRING v11: protein-protein association networks with increased coverage, supporting functional discovery in genome-wide experimental datasets. Nucleic Acids Res.

[29] Zhang Y, Chen K, Sloan SA, Bennett ML, Scholze AR, O'Keeffe S (2014). An RNA-sequencing transcriptome and splicing database of glia, neurons, and vascular cells of the cerebral cortex. J Neurosci.

[30] Livak KJ, Schmittgen TD (2001). Analysis of relative gene expression data using real-time quantitative PCR and the 2(−Delta Delta C(T)) method. Methods..

[31] Kercher L, Favara C, Striebel JF, LaCasse R, Chesebro B (2007). Prion protein expression differences in microglia and astroglia influence scrapie-induced neurodegeneration in the retina and brain of transgenic mice. J Virol.

[32] Young MD, Wakefield MJ, Smyth GK, Oshlack A (2010). Gene ontology analysis for RNA-seq: accounting for selection bias. Genome Biol.

[33] Schulte U, Thumfart JO, Klocker N, Sailer CA, Bildl W, Biniossek M (2006). The epilepsy-linked Lgi1 protein assembles into presynaptic Kv1 channels and inhibits inactivation by Kvbeta1. Neuron..

[34] Butz S, Okamoto M, Sudhof TC (1998). A tripartite protein complex with the potential to couple synaptic vesicle exocytosis to cell adhesion in brain. Cell..

[35] Vincenti JE, Murphy L, Grabert K, McColl BW, Cancellotti E, Freeman TC (2015). Defining the microglia response during the time course of chronic neurodegeneration. J Virol.

[36] Aguzzi A, Zhu C (2017). Microglia in prion diseases. J Clin Invest.

[37] Schaeren-Wiemers N, Schaefer C, Valenzuela DM, Yancopoulos GD, Schwab ME (1995). Identification of new oligodendrocyte- and myelin-specific genes by a differential screening approach. J Neurochem.

[38] Kim T, Fiedler K, Madison DL, Krueger WH, Pfeiffer SE (1995). Cloning and characterization of MVP17: a developmentally regulated myelin protein in oligodendrocytes. J Neurosci Res.

[39] Frank M (2000). MAL, a proteolipid in glycosphingolipid enriched domains: functional implications in myelin and beyond. Prog Neurobiol.

[40] Erdei A, Lukacsi S, Macsik-Valent B, Nagy-Balo Z, Kurucz I, Bajtay Z (2019). Non-identical twins: different faces of CR3 and CR4 in myeloid and lymphoid cells of mice and men. Semin Cell Dev Biol.

[41] Garnotel R, Rittie L, Poitevin S, Monboisse JC, Nguyen P, Potron G (2000). Human blood monocytes interact with type I collagen through alpha x beta 2 integrin (CD11c-CD18, gp150-95). J Immunol.

[42] Vaughan M, Moss J (1997). Activation of toxin ADP-ribosyltransferases by the family of ADP-ribosylation factors. Adv Exp Med Biol.

[43] Moss J, Vaughan M (1999). Activation of toxin ADP-ribosyltransferases by eukaryotic ADP-ribosylation factors. Mol Cell Biochem.

[44] Tomarev SI, Nakaya N (2009). Olfactomedin domain-containing proteins: possible mechanisms of action and functions in normal development and pathology. Mol Neurobiol.

[45] Carroll JA, Striebel JF, Rangel A, Woods T, Phillips K, Peterson KE (2016). Prion strain differences in accumulation of PrPSc on neurons and glia are associated with similar expression profiles of neuroinflammatory genes: comparison of three prion strains. PLoS Pathog.

[46] Christensen LB, Woods TA, Carmody AB, Caughey B, Peterson KE (2014). Age-related differences in neuroinflammatory responses associated with a distinct profile of regulatory markers on neonatal microglia. J Neuroinflammation.

[47] Butovsky O, Jedrychowski MP, Moore CS, Cialic R, Lanser AJ, Gabriely G (2014). Identification of a unique TGF-beta-dependent molecular and functional signature in microglia. Nat Neurosci.

[48] Kang W, Balordi F, Su N, Chen L, Fishell G, Hebert JM (2014). Astrocyte activation is suppressed in both normal and injured brain by FGF signaling. Proc Natl Acad Sci U S A.

[49] Betmouni S, Perry VH, Gordon JL (1996). Evidence for an early inflammatory response in the central nervous system of mice with scrapie. Neuroscience..

[50] Stichel CC, Luebbert H (2007). Inflammatory processes in the aging mouse brain: participation of dendritic cells and T-cells. Neurobiol Aging.

[51] Norden DM, Godbout JP (2013). Review: microglia of the aged brain: primed to be activated and resistant to regulation. Neuropathol Appl Neurobiol.

[52] Holtman IR, Raj DD, Miller JA, Schaafsma W, Yin Z, Brouwer N (2015). Induction of a common microglia gene expression signature by aging and neurodegenerative conditions: a co-expression meta-analysis. Acta Neuropathol Commun.

[53] Reichmann G, Schroeter M, Jander S, Fischer HG (2002). Dendritic cells and dendritic-like microglia in focal cortical ischemia of the mouse brain. J Neuroimmunol.

[54] Kamphuis W, Kooijman L, Schetters S, Orre M, Hol EM (1862). Transcriptional profiling of CD11c-positive microglia accumulating around amyloid plaques in a mouse model for Alzheimer’s disease. Biochim Biophys Acta.

[55] Wang Y, Cella M, Mallinson K, Ulrich JD, Young KL, Robinette ML (2015). TREM2 lipid sensing sustains the microglial response in an Alzheimer's disease model. Cell..

[56] Takada Y, Ye X, Simon S (2007). The integrins. Genome Biol.

[57] Davis GE (1992). The Mac-1 and p150,95 beta 2 integrins bind denatured proteins to mediate leukocyte cell-substrate adhesion. Exp Cell Res.

[58] Zhu C, Herrmann US, Li B, Abakumova I, Moos R, Schwarz P (2015). Triggering receptor expressed on myeloid cells-2 is involved in prion-induced microglial activation but does not contribute to prion pathogenesis in mouse brains. Neurobiol Aging.

[59] Tatzelt J, Maeda N, Pekny M, Yang SL, Betsholtz C, Eliasson C (1996). Scrapie in mice deficient in apolipoprotein E or glial fibrillary acidic protein. Neurology..

[60] Yun SP, Kam TI, Panicker N, Kim S, Oh Y, Park JS (2018). Block of A1 astrocyte conversion by microglia is neuroprotective in models of Parkinson’s disease. Nat Med.

[61] Lian H, Yang L, Cole A, Sun L, Chiang AC, Fowler SW (2015). NFkappaB-activated astroglial release of complement C3 compromises neuronal morphology and function associated with Alzheimer's disease. Neuron..

[62] Jin J, Smith MD, Kersbergen CJ, Kam TI, Viswanathan M, Martin K (2019). Glial pathology and retinal neurotoxicity in the anterior visual pathway in experimental autoimmune encephalomyelitis. Acta Neuropathol Commun.

[63] Schaeren-Wiemers N, Bonnet A, Erb M, Erne B, Bartsch U, Kern F (2004). The raft-associated protein MAL is required for maintenance of proper axon--glia interactions in the central nervous system. J Cell Biol.

[64] Bosio A, Binczek E, Stoffel W (1996). Functional breakdown of the lipid bilayer of the myelin membrane in central and peripheral nervous system by disrupted galactocerebroside synthesis. Proc Natl Acad Sci U S A.

[65] Schnaar RL, Suzuki A, Stanley P, Varki A, Cummings RD, Esko JD, Freeze HH, Stanley P, Bertozzi CR, Hart GW, Etzler ME (2009). Glycosphingolipids. Essentials of glycobiology.

[66] Brockschnieder D, Sabanay H, Riethmacher D, Peles E (2006). Ermin, a myelinating oligodendrocyte-specific protein that regulates cell morphology. J Neurosci.

[67] Zhang B, Cao Q, Guo A, Chu H, Chan YG, Buschdorf JP (2005). Juxtanodin: an oligodendroglial protein that promotes cellular arborization and 2′,3′-cyclic nucleotide-3′-phosphodiesterase trafficking. Proc Natl Acad Sci U S A.

[68] Dorandeu A, Wingertsmann L, Chretien F, Delisle MB, Vital C, Parchi P (1998). Neuronal apoptosis in fatal familial insomnia. Brain Pathol.

[69] Jesionek-Kupnicka D, Buczynski J, Kordek R, Liberski PP (1999). Neuronal loss and apoptosis in experimental Creutzfeldt-Jakob disease in mice. Folia Neuropathol.

[70] Jesionek-Kupnicka D, Kordek R, Buczynski J, Liberski PP (2001). Apoptosis in relation to neuronal loss in experimental Creutzfeldt-Jakob disease in mice. Acta Neurobiol Exp (Wars).

[71] Saa P, Harris DA, Cervenakova L (2016). Mechanisms of prion-induced neurodegeneration. Expert Rev Mol Med.

[72] Pignatelli M, Luna-Medina R, Perez-Rendon A, Santos A, Perez-Castillo A (2003). The transcription factor early growth response factor-1 (EGR-1) promotes apoptosis of neuroblastoma cells. Biochem J.

[73] Estus S, Zaks WJ, Freeman RS, Gruda M, Bravo R, Johnson EM (1994). Altered gene expression in neurons during programmed cell death: identification of c-Jun as necessary for neuronal apoptosis. J Cell Biol.

[74] Bossy-Wetzel E, Bakiri L, Yaniv M (1997). Induction of apoptosis by the transcription factor c-Jun. EMBO J.

[75] Coultas L, Terzano S, Thomas T, Voss A, Reid K, Stanley EG (2007). Hrk/DP5 contributes to the apoptosis of select neuronal populations but is dispensable for haematopoietic cell apoptosis. J Cell Sci.

[76] Ma C, Ying C, Yuan Z, Song B, Li D, Liu Y (2007). dp5/HRK is a c-Jun target gene and required for apoptosis induced by potassium deprivation in cerebellar granule neurons. J Biol Chem.

[77] Inohara N, Ding L, Chen S, Nunez G (1997). Harakiri, a novel regulator of cell death, encodes a protein that activates apoptosis and interacts selectively with survival-promoting proteins Bcl-2 and Bcl-X(L). EMBO J.

[78] Kim TK, Lee I, Cho JH, Canine B, Keller A, Price ND (2020). Core transcriptional regulatory circuits in prion diseases. Mol Brain.

[79] Carimalo J, Cronier S, Petit G, Peyrin JM, Boukhtouche F, Arbez N (2005). Activation of the JNK-c-Jun pathway during the early phase of neuronal apoptosis induced by PrP106-126 and prion infection. Eur J Neurosci.

[80] Stoka V, Turk V, Turk B (2007). Lysosomal cysteine cathepsins: signaling pathways in apoptosis. Biol Chem.

[81] Cataldo AM, Barnett JL, Berman SA, Li J, Quarless S, Bursztajn S (1995). Gene expression and cellular content of cathepsin D in Alzheimer’s disease brain: evidence for early up-regulation of the endosomal-lysosomal system. Neuron..

[82] Boland B, Campbell V (2004). Abeta-mediated activation of the apoptotic cascade in cultured cortical neurones: a role for cathepsin-L. Neurobiol Aging.

[83] Cataldo AM, Nixon RA (1990). Enzymatically active lysosomal proteases are associated with amyloid deposits in Alzheimer brain. Proc Natl Acad Sci U S A.

[84] Cataldo AM, Paskevich PA, Kominami E, Nixon RA (1991). Lysosomal hydrolases of different classes are abnormally distributed in brains of patients with Alzheimer disease. Proc Natl Acad Sci U S A.

[85] Thellung S, Corsaro A, Villa V, Simi A, Vella S, Pagano A (2011). Human PrP90-231-induced cell death is associated with intracellular accumulation of insoluble and protease-resistant macroaggregates and lysosomal dysfunction. Cell Death Dis.

[86] Doh-Ura K, Iwaki T, Caughey B (2000). Lysosomotropic agents and cysteine protease inhibitors inhibit scrapie-associated prion protein accumulation. J Virol.

[87] Caughey B (1993). Scrapie associated PrP accumulation and its prevention: insights from cell culture. Br Med Bull.

[88] Caughey B, Raymond GJ, Ernst D, Race RE (1991). N-terminal truncation of the scrapie-associated form of PrP by lysosomal protease(s): implications regarding the site of conversion of PrP to the protease-resistant state. J Virol.

[89] Magalhaes AC, Baron GS, Lee KS, Steele-Mortimer O, Dorward D, Prado MA (2005). Uptake and neuritic transport of scrapie prion protein coincident with infection of neuronal cells. J Neurosci.

[90] Hwang D, Lee IY, Yoo H, Gehlenborg N, Cho JH, Petritis B (2009). A systems approach to prion disease. Mol Syst Biol.

[91] Muth C, Schrock K, Madore C, Hartmann K, Fanek Z, Butovsky O (2017). Activation of microglia by retroviral infection correlates with transient clearance of prions from the brain but does not change incubation time. Brain Pathol.

