# Supplementary material for: RNA-seq and network analysis reveal unique glial gene expression signatures during prion infection
Source: Mol Brain. 2020 May 7;13:71. doi: 10.1186/s13041-020-00610-8 (PMC7206698; doi:10.1186/s13041-020-00610-8)
Supplement: Supplementary file 1 — Additional file 1: Table S1. C57BL/10 mice used throughout this study. [file 13041_2020_610_MOESM1_ESM.docx]

| **Additional File, Table S1.** C57BL/10 mice used throughout this study | | | | |  |  |
| --- | --- | --- | --- | --- | --- | --- |
| **Inocula** | **Treatment** | **Mouse #** | **Sex** | **Euthanized at** | **Microglia per field: cortex (% reduction)** | **Average % reduction** |
| 30ul 1% NBH IC | Untreated | B780-1 | M | 80 dpi | 98 |  |
|  |  | B780-2 | M | 80 dpi | 80 |  |
|  |  | B806-1 | M | 98 dpi | 76 | - |
|  |  | B806-2 | M | 98 dpi | 80 |  |
|  |  | B766-1 | F | 161 dpi | 120 |  |
|  |  | B766-2 | F | 161 dpi | 85 |  |
| 30ul 1% NBH IC | PLX5622 | B782-1 | M | 80 dpi | 12 (87%) |  |
|  |  | B782-2 | M | 80 dpi | 11 (88%) |  |
|  |  | B807-1 | M | 98 dpi | 19 (79%) | 90% |
|  |  | B807-2 | M | 98 dpi | 12 (87%) |  |
|  |  | B767-1 | F | 161 dpi | 1 (99%) |  |
|  |  | B767-2 | F | 161 dpi | 0 (100%) |  |
| 30ul 1% RML IC | Untreated | B772-1 | F | 80 dpi | 126 |  |
|  |  | B772-2 | F |  | 102 | - |
|  |  | B772-3 | F |  | 96 |  |
|  |  | B772-4 | F |  | 87 |  |
| 30ul 1% RML IC | PLX5622 | B770-1 | F | 80 dpi | 11 (89%) |  |
|  |  | B770-2 | F |  | 2 (98%) |  |
|  |  | B770-3 | F |  | 4 (96%) | 91% |
|  |  | B770-4 | F |  | 12 (88%) |  |
|  |  | B786-4 | M |  | 15 (85%) |  |
| 30ul 1% RML IC | Untreated | B768-1 | F | 100 dpi | 94 |  |
|  |  | B768-2 | F |  | 89 |  |
|  |  | B768-3 | F |  | 86 | - |
|  |  | B768-4 | F |  | 113 |  |
|  |  | B784-1 | M |  | 62 |  |
|  |  | B784-2 | M |  | 87 |  |
| 30ul 1% RML IC | PLX5622 | B771-1 | F | 100 dpi | 79 (11%) |  |
|  |  | B771-2 | F |  | 19 (79%) |  |
|  |  | B771-3 | F |  | 33 (63%) | 61% |
|  |  | B781-1 | M |  | 40 (55%) |  |
|  |  | B781-2 | M |  | 35 (61%) |  |
|  |  | B810-1 | F |  | 2 (98%) |  |
| 30ul 1% RML IC | Untreated | B765-3 | F | 153 dpi | ND |  |
|  |  | B765-4 | F | 157 dpi | ND |  |
|  |  | B769-1 | F | 153 dpi | 224 |  |
|  |  | B769-2 | F | 153 dpi | ND |  |
|  |  | B769-3 | F | 157 dpi | 237 |  |
|  |  | B787-1 | M | 160 dpi | ND |  |
|  |  | B787-2 | M | 160 dpi | 230 |  |
| 30ul 1% RML IC | PLX5622 | B764-1 | F | 117 dpi | ND |  |
|  |  | B773-2 | F | 117 dpi | 165 (28%) |  |
|  |  | B783-1 | M | 136 dpi | 142 (38%) |  |
|  |  | B783-2 | M | 136 dpi | 117 (49%) | 40% |
|  |  | B783-3 | M | 136 dpi | 107 (53%) |  |
|  |  | B783-4 | M | 142 dpi | 118 (49%) |  |
|  |  | B811-1 | F | 117 dpi | 154 (33%) |  |

M = male, F= female

NBH = Normal brain homogenate

RML = Strain RML prions

IC = intracerebrally

dpi = days post-inoculation

ND = not determined

% reduction with PLX5622 treatment is relative to the average microglia in untreated control mice as counted and reported in Carroll et al. (2018). *J Virol*, 92(15). doi:10.1128/JVI.00549-18
