# Supplementary material for: RNA-seq and network analysis reveal unique glial gene expression signatures during prion infection
Source: Mol Brain. 2020 May 7;13:71. doi: 10.1186/s13041-020-00610-8 (PMC7206698; doi:10.1186/s13041-020-00610-8)
Supplement: Supplementary file 2 — Additional file 2: Table S2. Gene expression during prion infection of the 23 transcripts typically increased in microglia associated with a neurodegenerative phenotype (MGnD). [file 13041_2020_610_MOESM2_ESM.docx]

| **Additional File, Table S2.** Gene expression during prion infection of the 23 transcripts typically increased in microglia associated with a neurodegenerative phenotype (MGnD). | | | | | | | | | |
| --- | --- | --- | --- | --- | --- | --- | --- | --- | --- |
|  | **80 dpi^†^** | |  | **100 dpi** | |  | **~157 dpi** | |  |
| **Gene** | **FC^‡^** | **P value** |  | **FC** | **P value** |  | **FC** | **P value** | **Description** |
| *Alcam* | -1.2 | 5.3x10^-2^ |  | 1.0 | 6.1x10^-1^ |  | 1.0 | 7.1x10^-1^ | activated leukocyte cell adhesion molecule |
| *Apoe* | -1.7 | 3.7x10^-2^ |  | 1.2 | 6.3x10^-2^ |  | **2.5** | **4.0x10^-10^** | apolipoprotein e |
| *Arg1* | 1.0 | 7.5x10^-1^ |  | 1.0 | 5.9x10^-1^ |  | 1.0 | 9.8x10^-1^ | arginase |
| *Axl* | 1.2 | 2.1x10^-2^ |  | 1.4 | 2.3x10^-4^ |  | **2.3** | **1.2x10^-6^** | AXL receptor tyrosine kinase |
| *Ccl2* | 1.1 | 1.3x10^-1^ |  | 1.2 | 1.3x10^-1^ |  | 1.5 | 3.3x10^-3^ | chemokine (C-C motif) ligand 2 |
| *Ccrl2* | 1.0 | 4.2x10^-1^ |  | 1.0 | 6.3x10^-1^ |  | 1.2 | 1.7x10^-1^ | chemokine (C-C motif) receptor-like 2 |
| *Cfp* | 1.0 | 5.3x10^-1^ |  | 1.1 | 2.3x10^-1^ |  | 1.0 | 7.4x10^-1^ | complement factor properdin |
| *Chi3l3/Chil3* | -1.1 | 1.9x10^-3^ |  | 1.0 | 4.8x10^-2^ |  | 1.1 | 1.4x10^-1^ | chitinase-like 3 |
| *Clec7a* | 1.6 | 4.7x10^-2^ |  | **2.9** | **2.6x10^-4^** |  | **7.9** | **2.7x10^-6^** | C-type lectin domain family 7, member a |
| *Csf1* | 1.1 | 1.8x10^-1^ |  | 1.3 | 5.5x10^-4^ |  | 1.8 | 3.4x10^-4^ | colony stimulating factor 1 |
| *Cxcl10* | **2.4** | **2.1x10^-3^** |  | **4.0** | **1.0x10^-5^** |  | **4.4** | **4.1x10^-6^** | chemokine (C-X-C motif) ligand 10 |
| *Cxcl16* | 1.1 | 5.9x10^-1^ |  | 1.6 | 2.0x10^-3^ |  | **2.9** | **3.2x10^-7^** | chemokine (C-X-C motif) ligand 16 |
| *Cxcr4* | -1.1 | 3.4x10^-1^ |  | 1.0 | 6.0x10^-1^ |  | 1.5 | 3.4x10^-4^ | chemokine (C-X-C motif) receptor 4 |
| *Cybb* | 1.0 | 9.4x10^-1^ |  | 1.3 | 6.7x10^-2^ |  | **3.6** | **6.5x10^-6^** | cytochrome b-245, beta polypeptide |
| *Fer1l3/Myof* | -1.1 | 3.5x10^-1^ |  | 1.0 | 6.6x10^-1^ |  | 1.5 | 3.1x10^-6^ | myoferlin |
| *Gas7* | -1.1 | 7.2x10^-1^ |  | -1.1 | 5.2x10^-1^ |  | -1.1 | 7.6x10^-1^ | growth arrest specific 7 |
| *Gpnmb* | 1.1 | 3.9x10^-1^ |  | 1.5 | 4.1x10^-4^ |  | **13.6** | **1.1x10^-11^** | glycoprotein (transmembrane) nmb |
| *Gpx3* | -1.3 | 8.6x10^-2^ |  | 1.2 | 1.6x10^-1^ |  | 1.1 | 2.9x10^-1^ | glutathione peroxidase 3 |
| *Ifi202b* | 1.0 | 4.5x10^-1^ |  | 1.0 | 2.7x10^-1^ |  | 1.0 | 4.0x10-^4^ | interferon activated gene 202B |
| *Itgax* | 1.4 | 6.1x10^-3^ |  | **2.8** | **1.5x10^-6^** |  | **9.4** | **5.1x10^-12^** | integrin alpha X |
| *Lag3* | **2.1** | **9.7x10^-4^** |  | **4.5** | **1.2x10^-6^** |  | **8.3** | **1.5x10^-9^** | lymphocyte-activation gene 3 |
| *Lgals3* | 1.0 | 8.6x10^-1^ |  | 1.3 | 9.7x10^-3^ |  | **4.0** | **2.4x10^-9^** | lectin, galactose binding, soluble 3 |
| *Lilrb4* | 1.0 | 9.6x10^-1^ |  | 1.1 | 4.3x10^-2^ |  | **3.6** | **3.8x10^-8^** | leukocyte immunoglobulin-like receptor, subfamily B, member 4A |
| *Msr1* | 1.0 | 2.3x10^-1^ |  | 1.0 | 6.1x10^-1^ |  | 1.4 | 3.2x10^-6^ | macrophage scavenger receptor 1 |
| *Siglec1* | 1.1 | 2.0x10^-2^ |  | 1.2 | 4.0x10^-3^ |  | 1.8 | 1.6x10^-8^ | sialic acid binding Ig-like lectin 1, sialoadhesin |
| *Spp1* | -1.3 | 8.1x10^-3^ |  | 1.1 | 7.1x10^-2^ |  | **5.0** | **2.2x10^-9^** | secreted phosphoprotein 1 |
| *Tlr2* | 1.1 | 2.9x10^-1^ |  | 1.9 | 2.2x10^-5^ |  | **3.6** | **3.9x10^-5^** | toll-like receptor 2 |

^†^ dpi = days post inoculation

^‡^ FC = fold change

Red bolded values denote genes increased ≥ 2.0-fold with p values ≤ 0.05 (5.0x10^-2^) in RML-infected mice.

Gray boxes indicate values that are increased between 1.5-fold and 1.9-fold with p values ≤ 0.05 (5.0x10^-2^) in RML-infected mice.
