# Supplementary material for: RNA-seq and network analysis reveal unique glial gene expression signatures during prion infection
Source: Mol Brain. 2020 May 7;13:71. doi: 10.1186/s13041-020-00610-8 (PMC7206698; doi:10.1186/s13041-020-00610-8)
Supplement: Supplementary file 3 — Additional file 3:Table S3. Gene expression during prion infection of the 68 transcripts typically decreased in microglia associated with a neurodegenerative phenotype (MGnD). [file 13041_2020_610_MOESM3_ESM.docx]

| **Additional File, Table S3.** Gene expression during prion infection of the 68 transcripts typically decreased in microglia associated with a neurodegenerative phenotype (MGnD). | | | | | | | | | |
| --- | --- | --- | --- | --- | --- | --- | --- | --- | --- |
|  | **80 dpi^†^** | |  | **100 dpi** | |  | **~157 dpi** | |  |
| **Gene** | **FC^‡^** | **P value** |  | **FC** | **P value** |  | **FC** | **P value** | **Description** |
| *Abcc3* | 1.1 | 3.8x10^-2^ |  | 1.2 | 6.3x10^-3^ |  | 1.8 | 1.1x10^-8^ | ATP-binding cassette, sub-family C (CFTR/MRP), member 3 |
| *Abi3* | 1.0 | 3.5x10^-1^ |  | 1.2 | 2.2x10^-2^ |  | 1.4 | 1.0x10^-6^ | ABI gene family, member 3 |
| *Arhgap5* | -1.2 | 1.5x10^-1^ |  | -1.6 | 6.9x10^-2^ |  | -1.3 | 9.4x10^-3^ | Rho GTPase activating protein 5 |
| *Atp8a2* | -1.0 | 8.8x10^-1^ |  | 1.0 | 9.9x10^-1^ |  | 1.1 | 4.1x10^-1^ | ATPase, aminophospholipid transporter-like, class I, type 8A, member 2 |
| *Basp1* | -1.1 | 6.4x10^-2^ |  | 1.0 | 7.6x10^-1^ |  | -1.2 | 4.6x10^-3^ | brain abundant, membrane attached signal protein 1 |
| *Bin1* | -1.1 | 3.5x10^-2^ |  | 1.0 | 8.3x10^-1^ |  | -1.0 | 2.7x10^-1^ | bridging integrator 1 |
| *Ccr5* | -1.5 | 3.0x10^-1^ |  | 1.0 | 9.8x10^-1^ |  | 1.1 | 8.4x10^-1^ | chemokine (C-C motif) receptor 5 |
| *Ckb* | -1.0 | 5.8x10^-1^ |  | 1.2 | 2.6x10^-2^ |  | 1.1 | 7.7x10^-2^ | creatine kinase, brain |
| *Cmklr1* | 1.3 | 1.5x10^-1^ |  | 1.1 | 5.6x10^-1^ |  | 1.3 | 2.9x10^-1^ | chemokine-like receptor 1 |
| *Crybb1* | -1.0 | 7.5x10^-1^ |  | 1.2 | 7.2x10^-2^ |  | 1.2 | 1.3x10^-2^ | crystallin, beta B1 |
| ***Csf1r*** | 1.2 | 1.6x10^-3^ |  | 1.5 | 1.0x10^-5^ |  | **2.4** | **7.1x10^-9^** | colony stimulating factor 1 receptor |
| *Cst3* | 1.1 | 5.3x10^-1^ |  | 1.6 | 1.6x10^-1^ |  | 1.8 | 2.5x10^-2^ | cystatin C |
| *Cttnbp2nl* | -1.0 | 9.0x10^-1^ |  | 1.2 | 4.1x10^-1^ |  | 1.9 | 5.8x10^-2^ | CTTNBP2 N-terminal like |
| *Cx3cr1* | 1.2 | 4.6x10^-3^ |  | -1.1 | 7.2x10^-1^ |  | 1.5 | 1.7x10^-1^ | chemokine (C-X3-C motif) receptor 1 |
| *Cxxc5* | 1.1 | 1.9x10^-1^ |  | 1.1 | 1.5x10^-1^ |  | 1.0 | 9.3x10^-1^ | CXXC finger 5 |
| ***Egr1*** | **-5.9** | **2.6x10^-2^** |  | **-6.1** | **4.7x10^-3^** |  | **-4.9** | **1.4x10^-2^** | early growth response 1 |
| *F11r* | -1.0 | 5.8x10^-1^ |  | 1.4 | 7.5x10^-4^ |  | 1.4 | 2.8x10^-4^ | F11 receptor |
| ***Fcrls*** | 1.5 | 1.6x10^-2^ |  | **2.2** | **2.5x10^-4^** |  | **4.9** | **5.4x10^-9^** | Fc receptor-like S, scavenger receptor |
| *Fgd2* | 1.2 | 4.2x10^-2^ |  | 1.3 | 7.4x10^-4^ |  | 1.7 | 4.6x10^-6^ | FYVE, RhoGEF and PH domain  containing 2 |
| *Fscn1* | -1.1 | 5.1x10^-1^ |  | 1.0 | 9.6x10^-1^ |  | -1.2 | 1.9x10^-1^ | fascin actin-bundling protein 1 |
| *Golm1* | 1.0 | 8.6x10^-1^ |  | 1.5 | 6.3x10^-2^ |  | 1.5 | 8.0x10^-2^ | golgi membrane protein 1 |
| *Gpr34* | 1.0 | 9.7x10^-1^ |  | -1.1 | 8.3x10^-1^ |  | 1.3 | 5.6x10^-1^ | G protein-coupled receptor 34 |
| *Gpr56/*  *Adgrg1* | 1.1 | 4.3x10^-1^ |  | 1.3 | 3.0x10^-2^ |  | 1.5 | 2.7x10^-3^ | adhesion G protein-coupled receptor G1 |
| *Gtf2h2* | -1.0 | 5.3x10^-1^ |  | 1.0 | 7.8x10^-1^ |  | -1.0 | 5.6x10^-1^ | general transcription factor II H,  polypeptide 2 |
| ***Hexb*** | 1.4 | 5.8x10^-3^ |  | 1.7 | 2.8x10^-5^ |  | **3.3** | **2.8x10^-8^** | hexosaminidase B |
| *Hpgds* | -1.1 | 5.0x10^-1^ |  | -1.2 | 6.8x10^-1^ |  | 1.6 | 1.0x10^-2^ | hematopoietic prostaglandin D synthase |
| *Il10ra* | 1.4 | 5.1x10^-2^ |  | 1.2 | 2.5x10^-1^ |  | 1.3 | 1.6x10^-1^ | interleukin 10 receptor, alpha |
| *Il10rb* | 1.0 | 8.2x10^-1^ |  | 1.1 | 1.5x10^-1^ |  | 1.4 | 6.9x10^-5^ | interleukin 10 receptor, beta |
| *Il21r* | 1.0 | 4.7x10^-1^ |  | 1.2 | 7.4x10^-3^ |  | 1.5 | 4.3x10^-6^ | interleukin 21 receptor |
| *Inpp4b* | -1.2 | 8.4x10^-3^ |  | -1.2 | 1.9x10^-2^ |  | -1.1 | 9.6x10^-2^ | inositol polyphosphate-4-phosphatase,  type II |
| *Itga6* | -1.0 | 6.1x10^-1^ |  | 1.0 | 9.0x10^-1^ |  | 1.3 | 5.2x10^-3^ | integrin alpha 6 |
| ***Jun*** | 2.6 | 2.8x10^-1^ |  | **9.1** | **7.5x10^-4^** |  | **11.5** | **1.3x10^-4^** | jun proto-oncogene |
| *Kctd12* | -1.2 | 8.4x10^-1^ |  | 1.5 | 4.5x10^-1^ |  | 1.3 | 7.5x10^-1^ | potassium channel tetramerisation domain containing 12 |
| *Lair1* | -1.1 | 3.5x10^-1^ |  | -1.1 | 3.0x10^-1^ |  | 1.4 | 7.1x10^-3^ | leukocyte-associated Ig-like receptor 1 (Lair1) |
| *Lrrc3* | 1.3 | 3.7x10^-1^ |  | 1.4 | 1.7x10^-1^ |  | -1.2 | 6.3x10^-1^ | leucine rich repeat containing 3 |
| *Ltc4s* | 1.1 | 5.0x10^-1^ |  | 1.1 | 3.3x10^-1^ |  | 1.8 | 1.3x10^-3^ | leukotriene C4 synthase |
| *Mafb* | 1.6 | 4.2x10^-1^ |  | -1.2 | 7.4x10^-1^ |  | 2.4 | 1.6x10^-1^ | v-maf musculoaponeurotic fibrosarcoma oncogene family, protein B |
| *Mef2a* | -1.6 | 3.4x10^-2^ |  | -1.5 | 7.4x10^-2^ |  | -1.2 | 1.7x10^-1^ | myocyte enhancer factor 2A |
| *Mertk* | 1.2 | 6.3x10^-3^ |  | 1.1 | 5.0x10^-1^ |  | 1.7 | 1.6x10^-7^ | c-mer proto-oncogene tyrosine kinase |
| *Nfkb1* | 1.1 | 4.0x10^-2^ |  | 1.2 | 1.2x10^-3^ |  | 1.3 | 4.8x10^-5^ | nuclear factor of kappa light polypeptide gene enhancer in B cells 1, p105 |
| *Nr3c1* | -1.1 | 5.6x10^-1^ |  | -1.1 | 3.5x10^-1^ |  | -1.1 | 1.2x10^-1^ | nuclear receptor subfamily 3, group C, member 1 |
| *Nrip1* | 1.6 | 1.3x10^-1^ |  | 1.1 | 6.6x10^-1^ |  | 1.1 | 8.6x10^-1^ | nuclear receptor interacting protein 1 |
| *Nuak1* | -1.1 | 5.9x10^-1^ |  | -1.2 | 9.2x10^-2^ |  | -1.1 | 2.1x10^-1^ | NUAK family, SNF1-like kinase, 1 |
| *Olfml3* | 1.5 | 1.3x10^-1^ |  | 1.3 | 7.2x10^-3^ |  | 1.9 | 7.5x10^-7^ | olfactomedin-like 3 |
| *Ophn1* | -1.0 | 6.8x10^-1^ |  | -1.2 | 3.0x10^-2^ |  | -1.0 | 4.1x10^-1^ | oligophrenin 1 |
| *P2ry12* | -1.2 | 2.8x10^-1^ |  | -1.4 | 1.4x10^-1^ |  | 1.3 | 1.9x10^-2^ | purinergic receptor P2Y, G-protein  coupled 12 |
| *P2ry13* | 1.2 | 7.2x10^-1^ |  | -1.1 | 8.1x10^-1^ |  | -1.3 | 6.3x10^-1^ | purinergic receptor P2Y, G-protein  coupled 13 |
| *Pde3b* | 1.1 | 3.9x10^-1^ |  | 1.1 | 1.6x10^-1^ |  | 1.2 | 7.3x10^-3^ | phosphodiesterase 3B, cGMP-inhibited |
| *Pla2g15* | 1.0 | 7.3x10^-1^ |  | 1.0 | 9.4x10^-1^ |  | 1.4 | 2.1x10^-6^ | phospholipase A2 |
| *Plxdc2* | -1.0 | 5.7x10^-1^ |  | 1.1 | 4.4x10^-1^ |  | 1.3 | 4.0x10^-4^ | plexin domain containing 2 |
| *Pmepa1* | 1.6 | 1.7x10^-1^ |  | 1.1 | 8.4x10^-1^ |  | -1.1 | 8.3x10^-1^ | prostate transmembrane protein, androgen induced 1 |
| *Rab3il1* | 1.1 | 1.1x10^-1^ |  | 1.3 | 4.0x10^-3^ |  | 1.4 | 2.1x10^-4^ | RAB3A interacting protein (rabin3)-like 1 |
| *Rap1gds1* | -1.4 | 6.3x10^-2^ |  | -1.1 | 6.2x10^-1^ |  | -1.1 | 3.0x10^-3^ | RAP1, GTP-GDP dissociation stimulator 1 |
| *Rgmb* | 1.3 | 3.3x10^-1^ |  | 1.0 | 8.3x10^-1^ |  | 1.0 | 8.7x10^-1^ | repulsive guidance molecule family  member B |
| *Rhob* | 1.1 | 1.2x10^-1^ |  | 1.2 | 4.2x10^-3^ |  | 1.1 | 2.2x10^-2^ | ras homolog family member B |
| *Sall1* | 1.0 | 8.9x10^-1^ |  | 1.1 | 2.4x10^-1^ |  | 1.4 | 1.6x10^-4^ | spalt like transcription factor 1 |
| *Scamp5* | 1.3 | 1.9x10^-1^ |  | 1.0 | 9.4x10^-1^ |  | -1.0 | 9.7x10^-1^ | secretory carrier membrane protein 5 |
| *Siglech* | 1.1 | 3.8x10^-1^ |  | 1.1 | 3.0x10^-1^ |  | 1.2 | 2.4x10^-1^ | sialic acid binding Ig-like lectin H |
| *Slc2a5* | 1.0 | 3.9x10^-1^ |  | 1.1 | 3.0x10^-2^ |  | 1.1 | 1.5x10^-1^ | solute carrier family 2 (facilitated glucose transporter), member 5 |
| *Slco2b1* | 1.4 | 2.4x10^-2^ |  | 1.4 | 1.6x10^-2^ |  | 1.9 | 4.2x10^-4^ | solute carrier organic anion transporter family, member 2b1 |
| *St3gal6* | -1.1 | 3.1x10^-1^ |  | -1.1 | 1.4x10^-1^ |  | 1.0 | 9.4x10^-1^ | ST3 beta-galactoside alpha-2,3-sialyltransferase 6 |
| ***Tgfb1*** | 1.1 | 1.8x10^-1^ |  | 1.6 | 1.1x10^-3^ |  | **2.7** | **1.6x10^-8^** | transforming growth factor, beta 1 |
| *Tgfbr1* | 1.3 | 3.7x10^-1^ |  | -1.3 | 5.1x10^-1^ |  | 1.4 | 1.8x10^-1^ | transforming growth factor, beta receptor I |
| *Tgfbr2* | -1.0 | 6.8x10^-1^ |  | 1.0 | 7.6x10^-1^ |  | 1.7 | 8.3x10^-3^ | transforming growth factor, beta receptor II |
| *Tjp1* | 1.0 | 5.5x10^-1^ |  | 1.0 | 7.9x10^-1^ |  | 1.2 | 2.0x10^-2^ | tight junction protein 1 |
| *Tmem119* | 1.1 | 4.8x10^-1^ |  | 1.4 | 3.3x10^-3^ |  | 1.2 | 7.4x10^-1^ | transmembrane protein 119 |
| *Usp2* | -1.1 | 4.0x10^-1^ |  | 1.1 | 2.2x10^-2^ |  | 1.0 | 6.7x10^-1^ | ubiquitin specific peptidase 2 |

^†^ dpi = days post inoculation

^‡^ FC = fold change

Red bolded values denote genes increased ≥ 2.0-fold with p values ≤ 0.05 (5.0x10^-2^) in RML-infected mice.

Blue bolded values denote genes decreased ≤ 2.0-fold with p values ≤ 0.05 (5.0x10^-2^) in RML-infected mice.

Gray boxes indicate values that are increased between 1.5-fold and 1.9-fold with p values ≤ 0.05 (5.0x10^-2^) in RML-infected mice.
