# Supplementary figures and images for: RNA-seq and network analysis reveal unique glial gene expression signatures during prion infection
Source: Mol Brain. 2020 May 7;13:71. doi: 10.1186/s13041-020-00610-8 (PMC7206698; doi:10.1186/s13041-020-00610-8)

Additional File Figure S1

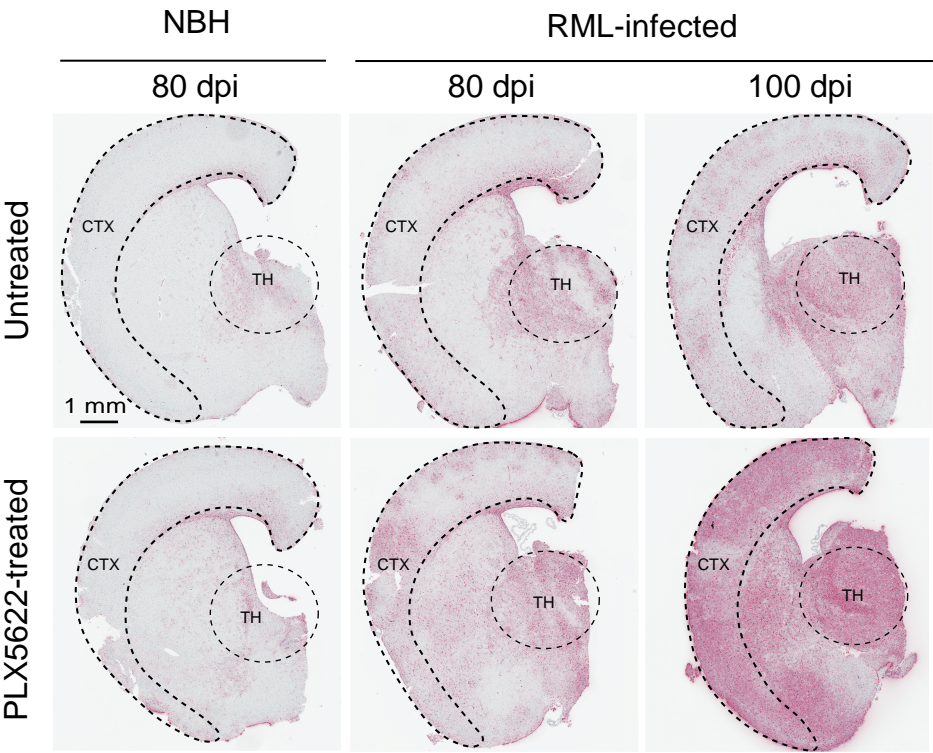

Supplement: Supplementary file 4 — Additional file 4:Figure S1. Representative immunohistochemical assessment of progressive astrogliosis in coronal sections from Untreated and PLX5622-treated mice. Mice were inoculated with either normal brain homogenate (NBH) or scrapie strain RML, and coronal sections from mice at 80 or 100 dpi were probed with antibodies against GFAP. Regions corresponding to the approximate location of the cerebral cortex (CTX) and thalamus (TH) are indicated or each. The scale for all representative images is indicated in the first panel. [file 13041_2020_610_MOESM4_ESM.pdf]
