# Supplementary material for: RNA-seq and network analysis reveal unique glial gene expression signatures during prion infection
Source: Mol Brain. 2020 May 7;13:71. doi: 10.1186/s13041-020-00610-8 (PMC7206698; doi:10.1186/s13041-020-00610-8)

Additional File Figure S2 page 1

A1 genes

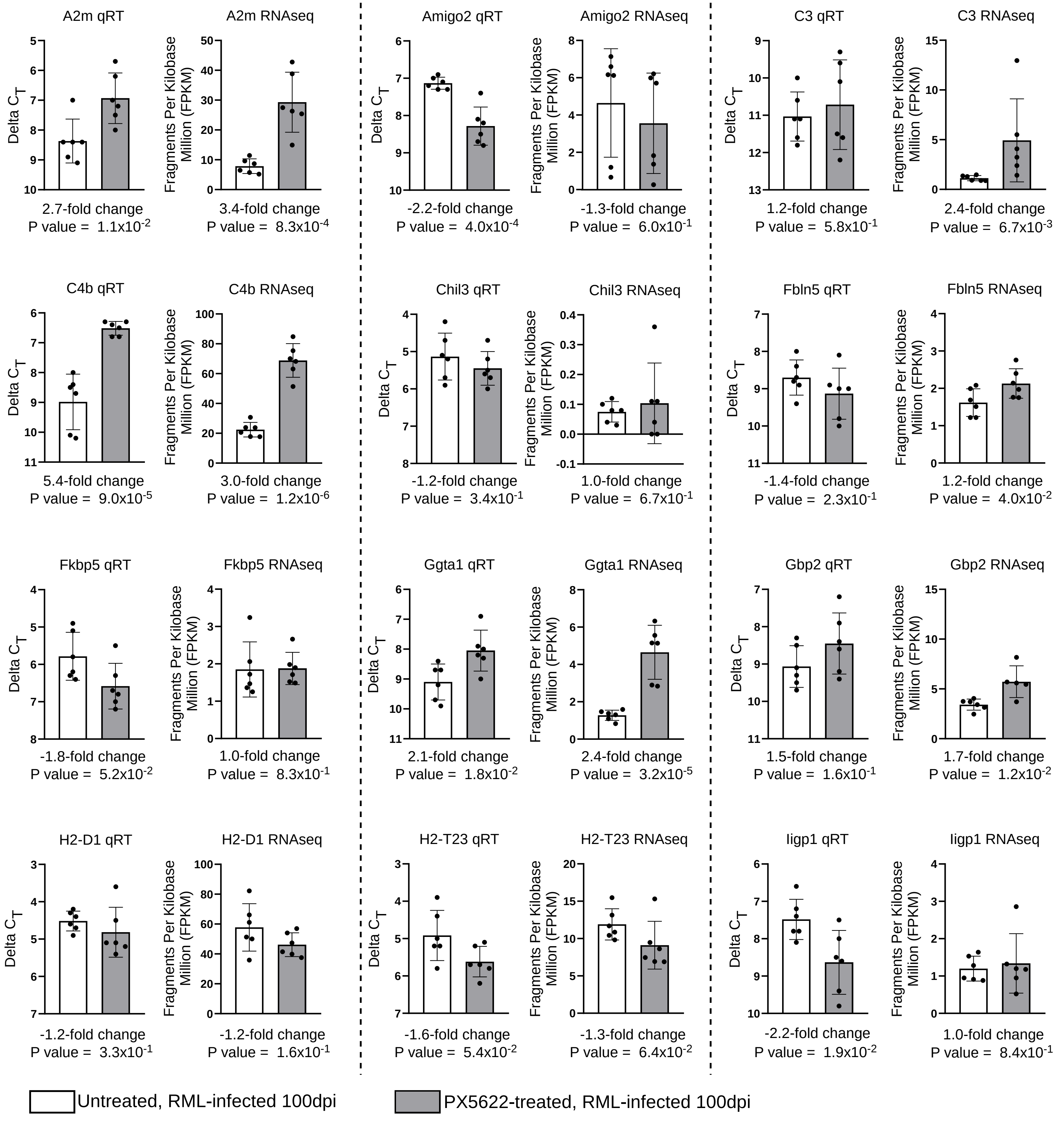

Additional File Figure S2 page 2

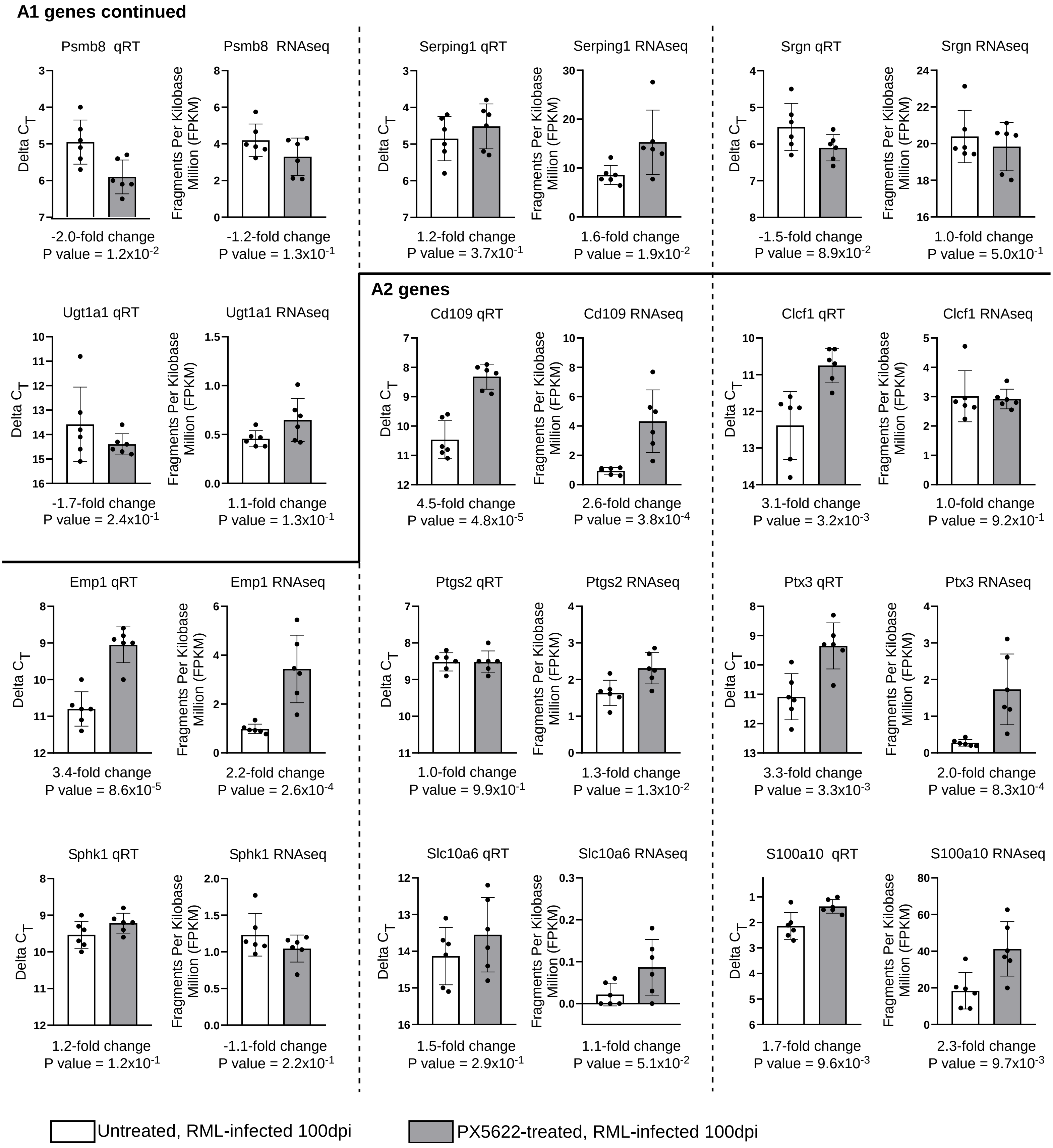

Additional File Figure S2 page 3

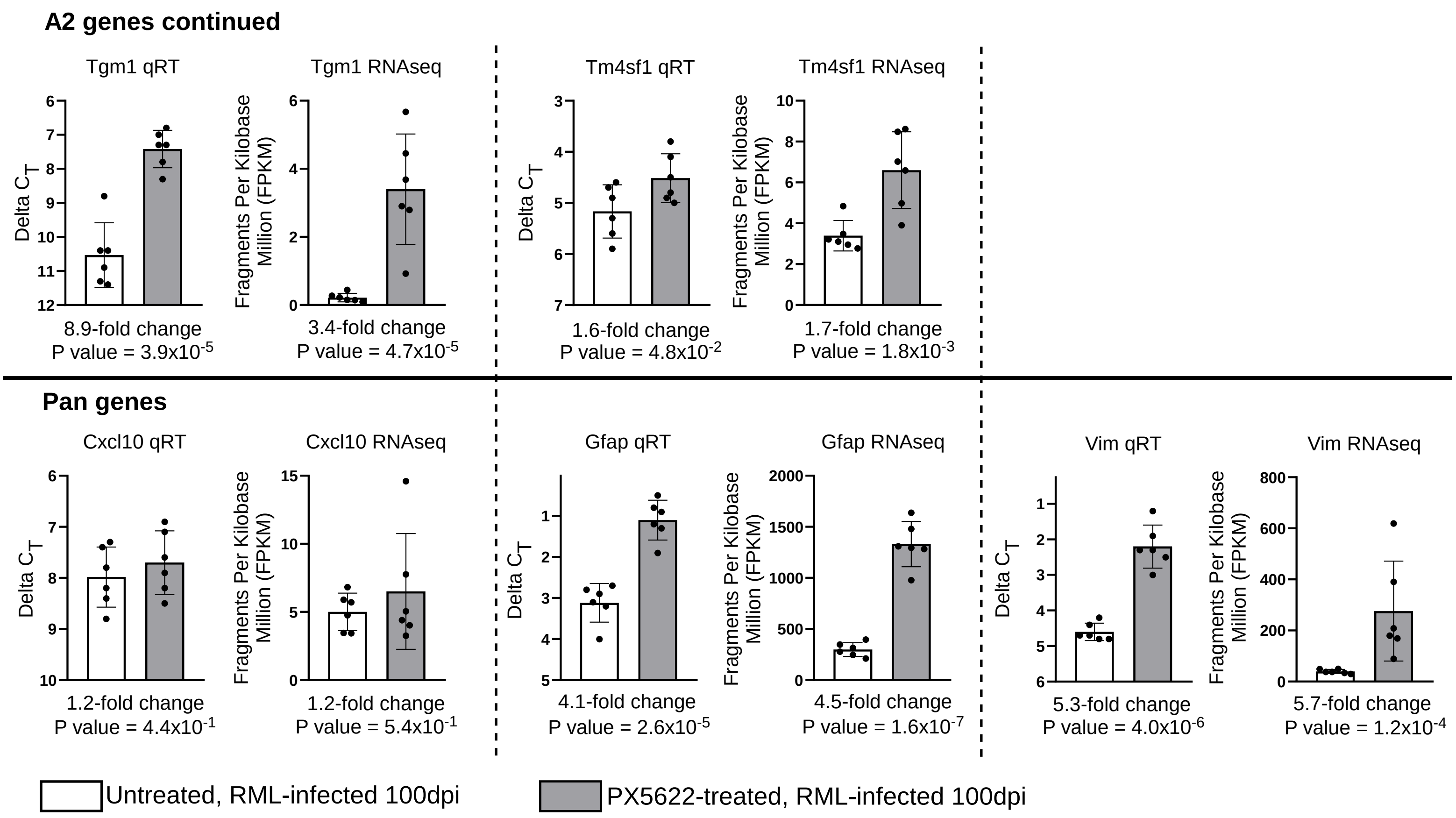

Supplement: Supplementary file 5 — Additional file 5:Figure S2. Expanded qRT-PCR analysis of Pan-, A1-, and A2-associated genes from RNA isolated from the brains of 100 dpi RML-infected mice. Mice that were treated with PLX5622 are grey columns and Untreated are white columns. The qRT-PCR results are presented as the Delta CT values. Also present is the RNA-seq data in Fragments Per Kilobase Million (FPKM) for each gene for comparison. Each dot represents the analysis of an individual mouse. The bars represent 1 standard deviation from the mean. P values and fold change of PXL5622 treatment relative to untreated are below each graph. [file 13041_2020_610_MOESM5_ESM.pdf]
